# Interactive Selection of Reference Sets in Multistage Bipolar Method

**DOI:** 10.3390/e28010054

**Published:** 2025-12-31

**Authors:** Maciej Nowak, Tadeusz Trzaskalik

**Affiliations:** Department of Operations Research, University of Economics in Katowice, 40-287 Katowice, Poland; tadeusz.trzaskalik@ue.katowice.pl

**Keywords:** multiple criteria decision making, reference point-based approach, Multistage Bipolar method, interactive approach, regional development planning, public management, mathematical economics

## Abstract

In this paper, the Multistage Bipolar method is developed. The paper presents a synthesis of three streams related to multiple criteria decision-making: the reference point-based approach, the interactive approach and multistage decision processes. A significant problem, the solution of which is a prerequisite for the application of the Multistage Bipolar method, is the determination of the sets of reference objects for subsequent stages. This paper addresses the question of how to utilize an interactive multi-criteria approach to select subsets of ‘good’ and ‘bad’ objects for each stage of the considered process, which the decision-maker will accept as sets of reference objects. Its objective is to propose an interactive procedure for generating these sets. The approach proposed in this paper is illustrated by the utilization of stage sets of reference points, generated via the proposed interactive procedure, within a mathematical model for resource allocation in a multistage regional development planning problem. The problem addressed constitutes a mathematical economics model, while simultaneously demonstrating that multi-criteria methods are widely applicable in management. Of fundamental importance here is the expenditure of public funds in a manner that yields maximum benefits for citizens.

## 1. Introduction

Multi-Criteria Decision Analysis (MCDA) is a rapidly developing branch of Operations Research. One way of developing multi-criteria methods is the utilization of reference points. This originated as Compromise Programming [1,2]. The reference point was the ideal point, defined as a point in the criterion space where all considered evaluation criteria for the decision problem achieved their optimal values. The goal of this approach was to identify the solution closest to the ideal solution using a predetermined distance metric. A similar method for determining the final solution is found in the highly popular Goal Programming approach [3], as well as in Wierzbicki’s Reference Point Method [4]. The reference point is no longer the ideal solution, but rather an arbitrary point in the criterion space. The next step involves the combined use of both ideal and anti-ideal points, as realized in the Bi-Reference Procedure [5] or the Fuzzy Bi-Reference Procedure [6].

It is worth referring to ideal point analysis as a family of multi-criteria methods, applied in psychometrics for attitude modeling [7] and in political science to position politicians relative to an ideal point on an ideological axis [8], as well as in decision sciences, where the TOPSIS method [9] is particularly prominent. Many variants and variations of this method have appeared [10]. A natural extension of the approach utilizing ideal and anti-ideal points, or another reference point indicated by the decision-maker, is the employment of a set of such points. One of the earliest papers in this domain is [11]. The Bipolar method was presented by Konarzewska-Gubała [12,13].

In the Bipolar method, two sets of reference points are considered: a set of ‘good’ points, possessing properties desired by the decision-maker, and a set of ‘bad’ points, which the decision-maker deems undesirable. Decision alternatives are not compared directly against each other, but rather against the reference points belonging to sets of ‘good’ and ‘bad’ objects using the ELECTRE methodology [14].

Another widely utilized method in multi-criteria optimization is the interactive approach. It assumes that the decision-maker is capable of providing information of a local nature—that is, information regarding a specific alternative or a small subset of decision alternatives. In a classical approach, information about the decision-maker’s preferences is obtained before the calculation procedure is launched, the purpose of which is to determine the solution to the problem. In contrast, in an interactive approach, this information is obtained step by step. In each iteration, the decision-maker is tasked with evaluating a specific decision alternative or a set of alternatives. The information thus obtained is then used to determine the next proposal, which is subsequently presented to the decision-maker. The procedure continues until a solution is found that the decision-maker considers satisfactory.

The first interactive methods were proposed in the 1970s and were utilized for solving multi-criteria linear or convex programming problems [15,16,17,18]. Their scope of application was subsequently extended to discrete problems—that is, those wherein the number of decision alternatives is finite and relatively small—including problems of multi-criteria decision-making under risk [19,20].

The third approach in the development of multi-criteria methods considered in this paper comprises multistage decision processes and discrete multi-criteria dynamic programming [21]. This approach employs a vector criterion function for the overall evaluation of the process, which is a composition of stage-wise criterion functions.

In this paper, the Multistage Bipolar method [22,23,24] is used. We consider a multistage decision process (with a finite number of stages) in which stage decisions (hereinafter referred to as stage alternatives) are evaluated with respect to criteria selected by the decision-maker. This method allows for overcoming a certain difficulty encountered when using Multi-Criteria Dynamic Programming. Decision-makers are often interested not only in the multistage values of criteria; stage values are often equally or even more important to them than the multistage values.

In the Multistage Bipolar method, these difficulties are overcome by utilizing two stage sets of reference points—hereinafter referred to as sets of reference objects—at each stage to compare the stage criterion values of the considered stage decisions (hereinafter referred to as stage alternatives). These include a set of ‘good’ objects defining stage values desired by the decision-maker and a set of ‘bad’ reference objects defining undesirable values. These sets may differ in subsequent stages of the process.

A significant problem, the solution of which is a prerequisite for the application of the Multistage Bipolar method, is the determination of the sets of reference objects for subsequent stages. Both existing data, such as stock market company ratings [25], and expert opinions, which form the basis for generating these sets, can be used for this purpose. In both cases, it may turn out that the size of the obtained sets is excessive, necessitating their reduction by the decision-maker.

This paper addresses the question of how to utilize multi-criteria approach to select subsets of ‘good’ and ‘bad’ objects for each stage of the process. It is assumed that a set of candidates for reference objects is available. The aim of the procedure is to select those among them that the decision-maker accepts as ‘good’ or ‘bad’ ones. To solve this problem, we use an interactive approach. The proposed objects are presented to the decision-maker. The decision-maker’s decision to accept or reject an object as ’good’/’bad’ is then used to determine the next proposals.

The approach proposed in this paper is illustrated by the utilization of stage sets of reference points, generated via the proposed interactive procedure, within a mathematical model for resource allocation in a multistage regional development planning problem. This is one of the fundamental problems of public management—how to allocate available resources in order to improve citizens’ living conditions as much as possible. We employ synthetic indicators to structure the expenditures related to the economic, social and environmental order in an example region in order to best achieve the sustainable development goals defined by the decision-maker.

The Multistage Bipolar method has never been used in the field of regional planning. Ref. [26] is highly relevant to our research as it bridges the gap between theoretical control models and their practical application in strategic planning. The author provides a formalized framework for multistage decision-making that effectively incorporates uncertainty and evolving socio-economic priorities.

Resource allocation is a frequently discussed dynamic optimization problem in the operational research literature [27]. It can be formulated as follows: a specific resource is given, and it should be allocated to maximize the total effect. We will use this approach to model the problem of shaping a regional sustainable development plan, which aims to equalize intra-regional disparities. Development goals apply to all aspects of life, referred to as economic order, social order and environmental order, respectively [28,29,30,31].

The assessment of sustainable development is, on the one hand, multi-criteria in nature and, at the same time, involves the use of aggregated measures [32]. The most common way to consider the problem is to calculate synthetic measures encompassing various factors. There are numerous studies comparing regional development using these integrated measures. Their goal is most often to rank regions and position them relative to others [33,34]. We will further deal with synthetic measures of regional development, but we will use them not for interregional comparisons but instead for shaping the expenditure related to the economic, social and environmental order in an exemplary region.

This paper consists of six chapters. Following the introduction in Section 1, the Multistage Bipolar method is briefly presented in Section 2. Section 3 describes the proposed interactive procedure subsequently used for generating ‘good’ and ‘bad’ objects in the successive stages of the Multistage Bipolar method. A numerical illustration of the proposed procedure is presented in Section 4 (Example 1), while its application in constructing a long-term sustainable development plan is found in Section 5 (Example 2). Conclusions are provided in Section 6.

## 2. Multistage Bipolar Procedure

The Multistage Bipolar Procedure is a procedure that enables the sorting of multistage decision alternatives, their ranking and the selection of the best multistage alternative. Below, we present a brief description of this procedure based on previous works [22,23,24]. We consider a multistage decision process consisting of *T* stages. At each stage, we consider sets of stage alternatives $A_{t}$ (*t* = 1, …, *T*), composed of stage alternatives. These are evaluated using *K* stage criterion functions, $f_{t}^{k}$ (*k* = 1, …, *K*). We assume that for each stage, the following have been defined:

- The stage sets of good reference objects, $G_{t}$ (*t* = 1, …, *T*), consisting of objects $g_{t}$;
- The stage sets of bad reference objects, $B_{t}$ (*t* = 1, …, *T*), consisting of objects $b_{t}$;
- Weights, $w_{t}^{k}$, for the stage criteria (*t* = 1, …, *T*, *k* = 1, …, *K*).

At each stage, the reference set of good objects is disjoint from the reference set of bad objects.

Stage alternatives are not compared directly with one another; instead, their position is determined relative to the stage sets of reference objects $G_{t}$ and $B_{t}$.

By combining these stage alternatives, we obtain the set of multistage alternatives, A. Each multistage alternative a∈A is a combination of different stage alternatives from different stages and is in the following form:

(1) $$a=\left(a_{1},\ldots ,a_{T}\right).$$

We determine the position of multistage alternatives by calculating, for each multistage alternative, the multistage failure avoidance degree and the multistage success achievement degree defined for the classic (one-step) Bipolar method by Konarzewska-Gubała [12,13].

Next, we sort the multistage alternatives into three predefined classes: **A***^H^* (class number 1), **A***^M^* (class number 2) and **A***^L^* (class number 3). Each multistage alternative from class **A***^H^* is superior to any other multistage alternative from classes **A***^M^* and **A***^L^*, whereas each multistage alternative belonging to class **A***^M^* is superior to any multistage alternative belonging to class **A***^L^*. Precise definitions will be introduced during the presentation of Example 2.

To determine the best multistage alternative, we identify the non-empty class with the lowest number. We rank the multistage alternatives using a quality index, starting with the multistage alternative for which this index has the highest value. A detailed description of this procedure can be found in [22,23,24].

To sum up, the Multistage Bipolar Procedure consists of the following 5 steps.

Step 1: Stage alternatives are compared with reference objects. Well-known procedures, such as ELECTRE, PROMETHEE and AHP (see [35]), can be employed.

Step 2: The position of each stage alternative with respect to the bipolar stage reference system is established.

Step 3: Relationships within the set of multistage alternatives are established.

Step 4: Multistage alternatives are assigned to predefined classes.

Step 5: The final solution is selected from the multistage alternatives belonging to the class with the lowest number.

## 3. Selection of Good and Bad Reference Objects—Interactive Procedure

### 3.1. Introductory Remarks

The purpose of the procedure is to enable the decision-maker to identify $G_{t}$ and $B_{t}$ for each stage. Initially, sets of candidates for good and bad objects are created. They can be generated in various ways, for example, based on historical data. In this paper, we assume that, for each stage, the sets of candidates are generated randomly.

In order for the procedure to run smoothly, candidates for good and bad objects should be presented to the decision-maker in the appropriate order. For this reason, we ask them to define a hierarchy of criteria for each stage, starting with the criterion that they consider most important. We assume that the sets of good and bad objects should be appropriately diversified, i.e., they should not contain only objects that are highly rated according to the most important criterion. For this reason, during the procedure, the objects with the best values for the subsequent criteria are sequentially proposed to the decision-maker.

In ensuring that the procedure is well received by the decision-maker, they should not be faced with the need to evaluate an object that, based on previously expressed opinions, should be automatically eliminated from further analysis. For this reason, when determining the set of good objects, we do not present the decision-maker with an object that is dominated by a previously analyzed one. We define the dominance relation in terms of the Pareto principle [36,37], which means that object *c* dominates object *c*’ if *c* is not worse than *c*’ with respect to any criterion and is better than *c*’ with respect to at least one criterion.

Similarly, when determining the set of bad objects, we do not propose to the decision-maker an object that dominates the one that was previously proposed. This approach also ensures that both sets will consist of non-dominated objects, which guarantees that the generated sets of good and bad objects will be sufficiently diverse.

Taking into account the assumptions of the Bipolar method, it can be assumed that the sets of good and bad objects should contain at least three elements. The maximum size of these sets is not predetermined. In the procedure presented here, it is assumed that the number of good and bad objects is determined either by the decision-maker or by the analyst assisting them in solving the problem. Both sets should be sufficiently diversified. In real-life applications [25], the number of good and bad objects usually does not exceed 10. Adding more usually does not improve the results. However, as a result of the procedure, it may turn out that it was not possible to identify the number of objects that was initially assumed. If this number is not less than the accepted minimum (e.g., 3 objects), the sets obtained can be accepted and then used in the Bipolar method. Otherwise, the procedure for determining good and bad objects should be repeated for the same or a new set of candidates.

### 3.2. Description of the Procedure

Notation:

$C_{t}$—a stage set of candidates for good or bad objects in stage *t*;
$n_{t}^{G}$—the required number of good objects in stage *t*;
$n_{t}^{B}$ —the required number of bad objects in stage *t*;
$m_{t}^{G}$—the current number of good objects in stage *t*;
$m_{t}^{B}$—the current number of bad objects in stage *t*;
${\tilde{C}}_{t}$—the set of objects that, in the current iteration, can still be proposed to the decision-maker as a good or bad object;
$R_{t}$—the set of objects that have been suggested to the decision-maker as good or bad objects and have not been accepted by them;
$h_{t}^{i}$—the position of criterion *i* in the hierarchy of criteria at stage *t*.

Initial phase:

Step I1: Determine the values of $n_{t}^{G}$ and $n_{t}^{B}$.
Step I2: Assume $G_{t}∶=\emptyset$, $B_{t}∶=\emptyset$, $m_{t}^{G}∶=0$, $m_{t}^{B}∶=0$.
Step I3: Define the hierarchy of criteria—specify the value of $h_{t}^{i}$ for *i* = 1, …, *K*.

Identification of the stage set of good objects:

Step G1: Assume ${\tilde{C}}_{t}∶=C_{t}$, $R_{t}≔\emptyset ,s=1$.
Step G2: Identify the criterion for which $h_{t}^{i}=\;s$, and assume l=i.
Step G3: Identify ${\bar{c}}_{t}\in {\tilde{C}}_{t}$ for which $f_{t}^{l}$ is maximized; if there is more than one such object, select one of them using a hierarchical approach, applying the hierarchy defined by the decision-maker.
Step G4: If $R_{t}=\emptyset$ and $G_{t}=\emptyset$, proceed to Step G6.
Step G5: If ${\bar{c}}_{t}$ is dominated by at least one $c_{t}\in R_{t}\cup G_{t},\;$then proceed to Step G8.
Step G6: Present ${\bar{c}}_{t}$ and $f_{t}^{k}\left({\bar{c}}_{t}\right)$ for *k* = 1, …, *K* to the decision-maker.
Step G7: Ask the decision-maker whether ${\bar{c}}_{t}$ can be considered a good object. If the answer is ‘yes’, proceed to Step G9.
Step G8: Add ${\bar{c}}_{t}$ to $R_{t}$ and remove it from ${\tilde{C}}_{t}$:
  (2) $$R_{t}∶=R_{t}\cup \left\{{\bar{c}}_{t}\right\}$$
  (3) $${\tilde{C}}_{t}∶={\tilde{C}}_{t}\backslash \left\{{\bar{c}}_{t}\right\}.$$
  and proceed to Step G3.
Step G9: Add ${\bar{c}}_{t}$ to $G_{t}$ and remove it from ${\tilde{C}}_{t}$:
  (4) $$G_{t}∶=G_{t}\cup \left\{{\bar{c}}_{t}\right\}$$
  (5) $${\tilde{C}}_{t}∶={\tilde{C}}_{t}\backslash \left\{{\bar{c}}_{t}\right\},$$
  (6) $$m_{t}^{G}∶=m_{t}^{G}+1.$$
Step G10: If $m_{t}^{G}=n_{t}^{G}$ or ${\tilde{C}}_{t}=\emptyset$, the identification of good objects is complete; go to the phase Identification of the stage set of bad objects.
Step G11: Assume s∶=s+1. If s>K, assume s∶=1. Proceed to Step G2.

**Figure 1:**
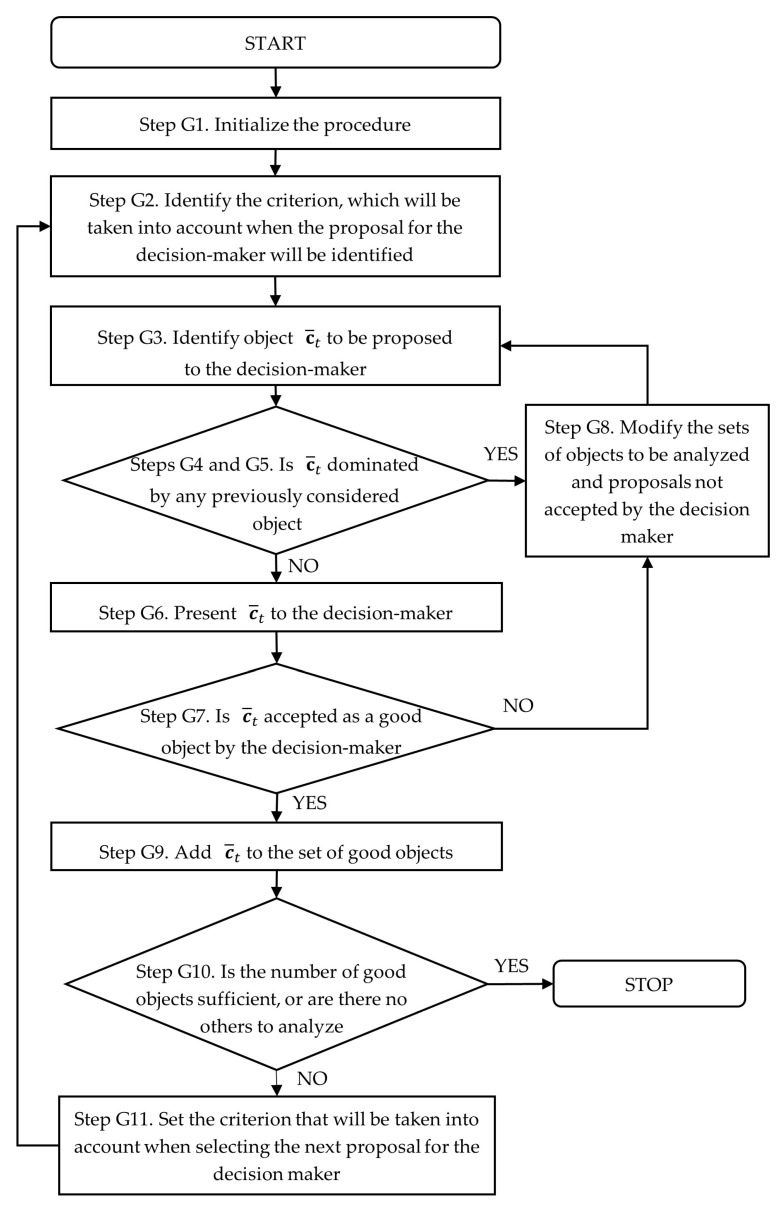
Flowchart of the procedure of good-object identification.

The flowchart of the procedure of good-object identification is presented in Figure 1. The identification of bad objects is very similar to that of good objects, except that in each case, the decision-maker is presented with an object that has a very low value for each of the criteria considered in turn.

Identification of the stage set of bad objects:

Step B1: Assume ${\tilde{C}}_{t}∶=C_{t}\backslash G_{t}$, $R_{t}≔\emptyset ,s=1$.
Step B2: Identify the criterion for which $h_{t}^{i}=\;s$, and assume l=i.
Step B3: Identify ${\bar{c}}_{t}\in {\tilde{C}}_{t}$ for which $f_{t}^{l}$ is minimized; if there is more than one such object, select one of them using a hierarchical approach, applying the hierarchy defined by the decision-maker.
Step B4: If $R_{t}=\emptyset$ and $B_{t}=\emptyset$, proceed to Step B6.
Step B5: If ${\bar{c}}_{t}$ dominates at least one $c_{t}\in R_{t}\cup B_{t},\;$then proceed to Step B8.
Step B6: Present ${\bar{c}}_{t}$ and $f_{t}^{k}\left({\bar{c}}_{t}\right)$ for *k* = 1, …, *K* to the decision-maker.
Step B7: Ask the decision-maker whether ${\bar{c}}_{t}$ can be considered a bad object. If the answer is ‘yes’, proceed to Step B9.
Step B8: Add ${\bar{c}}_{t}$ to $R_{t}$ and remove it from ${\tilde{C}}_{t}$:
  (7) $$R_{t}∶=R_{t}\cup \left\{{\bar{c}}_{t}\right\}$$
  (8) $${\tilde{C}}_{t}∶={\tilde{C}}_{t}\backslash \left\{{\bar{c}}_{t}\right\}.$$
  and proceed to Step B3.
Step B9: Add ${\bar{c}}_{t}$ to $B_{t}$ and remove it from ${\tilde{C}}_{t}$:
  (9) $$B_{t}∶=B_{t}\cup \left\{{\bar{c}}_{t}\right\}$$
  (10) $${\tilde{C}}_{t}∶={\tilde{C}}_{t}\backslash \left\{{\bar{c}}_{t}\right\},$$
  (11) $$m_{t}^{B}∶=m_{t}^{B}+1.$$
Step B10: If $m_{t}^{B}=n_{t}^{B}$ or ${\tilde{C}}_{t}=\emptyset$, the identification of bad objects is complete.
Step B11: Assume s∶=s+1. If s>K, assume s∶=1. Proceed to Step B2.

## 4. Numerical Illustration

The artificial sets of candidates for good and bad objects were generated randomly and are presented in Appendix A. Below, it is presented in detail how the sets of good and bad objects were generated for stage *t* = 1.

Initial phase:

Step I1: The following values were assumed for the number of good and bad objects: $n_{1}^{G}=3$, $n_{1}^{B}=3$.
Step I2: $G_{1}∶=\emptyset$, $B_{1}∶=\emptyset$, $m_{1}^{G}∶=0$ and $m_{1}^{B}∶=0$.
Step I3: The hierarchy of criteria was defined as $h_{1}^{1}=1$, $h_{1}^{2}=2$, $h_{1}^{3}=3$—the first criterion was the most important, the next was the second criterion and criterion no. 3 was the least important.

Identification of the stage set of good objects for stage *t* = 1:

Step G1: ${\tilde{C}}_{t}∶=C_{t}$, $R_{t}≔\emptyset ,s=1$.
Step G2: As $h_{1}^{i}=\;1$ for i=1, l=1.
Step G3: The object that achieved the highest score for the first criterion was $c_{67}$.
Step G4: As $R_{1}=\emptyset$ and $G_{1}=\emptyset$, the procedure proceeded to Step G6.
Step G6: Object $c_{67}$ was presented to the decision-maker: $f_{1}^{1}\left(c_{67}\right)=100$, $f_{1}^{2}\left(c_{67}\right)=10$ and $f_{1}^{3}\left(c_{67}\right)=59$.
Step G7: The decision-maker stated that, due to the insufficient value of the second criterion, $c_{67}$ could not be considered a good object.
Step G8: $c_{67}$ was added to $R_{1}$ and removed from ${\tilde{C}}_{1}$: $R_{1}∶=R_{1}\cup \left\{c_{67}\right\}$, ${\tilde{C}}_{1}∶={\tilde{C}}_{1}\backslash \left\{c_{67}\right\}$; the procedure proceeded to Step G3.
Step G3: There were two objects in ${\tilde{C}}_{1}$ that achieved the highest value of the first criterion, equal to 99: $c_{62}$ and $c_{85}$; since criterion no. 2 was the second most important according to the hierarchy defined by the decision-maker, we selected $c_{62}$ as a candidate for a good object: $f_{1}^{2}\left(c_{62}\right)=85$ vs. $f_{1}^{2}\left(c_{85}\right)=37$.
Step G4: As $R_{1}\neq \emptyset$, the procedure proceeded to Step G5.
Step G5: As $c_{62}$ was not dominated by $c_{67}$ (the only object in $R_{1}$), the procedure proceeded to Step G6.
Step G6: Object $c_{62}$ was presented to the decision-maker: $f_{1}^{1}\left(c_{62}\right)=99$, $f_{1}^{2}\left(c_{62}\right)=85$ and $f_{1}^{3}\left(c_{62}\right)=52$.
Step G7: The decision-maker accepted $c_{62}$ as a good object; the procedure proceeded to Step G9.
Step G9: $c_{62}$ was added to $G_{1}$ and removed from ${\tilde{C}}_{1}$: $G_{1}∶=G_{1}\cup \left\{c_{62}\right\}$, ${\tilde{C}}_{1}∶={\tilde{C}}_{1}\backslash \left\{c_{62}\right\}$ and $m_{1}^{G}=1$.
Step G10: As $m_{1}^{G}<n_{1}^{G}$ and ${\tilde{C}}_{1}\neq \emptyset$, the identification of good objects was continued.
Step G11: s∶=2; the procedure proceeded to Step G2.
Step G2: As $h_{1}^{i}=\;2$ for i=2, l=2.
Step G3: The object that achieved the highest score for the second criterion was $c_{53}$.
Step G4: As $R_{1}\neq \emptyset \;and\;G_{1}\neq \emptyset$, the procedure proceeded to Step G5.
Step G5: As $c_{53}$ was neither dominated by $c_{67}$ (the only object in $R_{1}$), nor by $c_{62}$ (the only object in $G_{1}$), the procedure proceeded to Step G6.
Step G6: Object $c_{53}$ was presented to the decision-maker: $f_{1}^{1}\left(c_{53}\right)=38$, $f_{1}^{2}\left(c_{53}\right)=100$ and $f_{1}^{3}\left(c_{53}\right)=5$.
Step G7: The decision-maker stated that, due to the insufficient value of the third criterion, $c_{53}$ could not be considered a good object.
Step G8: $c_{53}$ was added to $R_{1}$ and removed from ${\tilde{C}}_{1}$: $R_{1}∶=R_{1}\cup \left\{c_{53}\right\}$, ${\tilde{C}}_{1}∶={\tilde{C}}_{1}\backslash \left\{c_{53}\right\}$; the procedure proceeded to Step G3.
Step G3: There were two objects in ${\tilde{C}}_{1}$ achieving the highest value of the second criterion equal to 98: $c_{17}$ and $c_{35}$; taking into account the hierarchy of criteria, $c_{35}$ was selected as a candidate for a good object.
Step G4: As $R_{1}\neq \emptyset \;and\;G_{1}\neq \emptyset$, the procedure proceeded to Step G5.
Step G5: Object $c_{35}$ was dominated by $c_{53}\in R_{1}:f_{1}^{1}\left(c_{35}\right)=34<f_{1}^{1}\left(c_{53}\right)=38$, $f_{1}^{2}\left(c_{35}\right)=98<f_{1}^{2}\left(c_{53}\right)=100$, $f_{1}^{3}\left(c_{35}\right)=1<f_{1}^{3}\left(c_{53}\right)=5$; thus, the procedure proceeded to Step G8.
Step G8: $c_{35}$ was added to $R_{1}$ and removed from ${\tilde{C}}_{1}$: $R_{1}∶=R_{1}\cup \left\{c_{35}\right\}$, ${\tilde{C}}_{1}∶={\tilde{C}}_{1}\backslash \left\{c_{35}\right\}$; the procedure proceeded to Step G3.
Step G3: The second object for which the second criterion was equal to 98 ($c_{17})$ was selected as a candidate for a good object.
Step G4: As $R_{1}\neq \emptyset \;and\;G_{1}\neq \emptyset$, the procedure proceeded to Step G5.
Step G5: Object $c_{17}$ was dominated by $c_{53}\in R_{1}:f_{1}^{1}\left(c_{17}\right)=5<f_{1}^{1}\left(c_{53}\right)=38$, $f_{1}^{2}\left(c_{17}\right)=98<f_{1}^{2}\left(c_{53}\right)=100$, $f_{1}^{3}\left(c_{17}\right)=4<f_{1}^{3}\left(c_{53}\right)=5$; thus, the procedure proceeded to Step G8.
Step G8: $c_{17}$ was added to $R_{1}$ and removed from ${\tilde{C}}_{1}$: $R_{1}∶=R_{1}\cup \left\{c_{17}\right\}$, ${\tilde{C}}_{1}∶={\tilde{C}}_{1}\backslash \left\{c_{17}\right\}$; the procedure proceeded to Step G3.
Step G3: The object that achieved the highest score for the second criterion was $c_{6}$.
Step G4: As $R_{1}\neq \emptyset \;and\;G_{1}\neq \emptyset$, the procedure proceeded to Step G5.
Step G5: As $c_{6}$ was neither dominated by $c_{62}$ (the only object in $G_{1}$), nor by objects in $B_{1}$($c_{67},c_{53},c_{35},c_{17})$, the procedure proceeded to Step G6.
Step G6: Object $c_{6}$ was presented to the decision-maker: $f_{1}^{1}\left(c_{6}\right)=85$, $f_{1}^{2}\left(c_{6}\right)=95$, $f_{1}^{3}\left(c_{6}\right)=13$.
Step G7: The decision-maker accepted $c_{6}$ as a good object; the procedure proceeded to Step G9.
Step G9: $c_{6}$ was added to $G_{1}$ and removed from ${\tilde{C}}_{1}$: $G_{1}∶=G_{1}\cup \left\{c_{6}\right\}$, ${\tilde{C}}_{1}∶={\tilde{C}}_{1}\backslash \left\{c_{6}\right\}$, $m_{1}^{G}=2$.
Step G10: As $m_{1}^{G}<n_{1}^{G}$ and ${\tilde{C}}_{1}\neq \emptyset$, the identification of good objects was continued.
Step G11: s∶=3; procedure proceeded to Step G2.
Step G2: As $h_{1}^{i}=\;3$ for i=3, l=3.
Step G3: The object that achieved the highest score for the second criterion was $c_{54}$.
Step G4: As $R_{1}\neq \emptyset \;and\;G_{1}\neq \emptyset$, the procedure proceeded to Step G5.
Step G5: As $c_{54}$ was neither dominated by objects from $G_{1}$ ($c_{62},c_{6}$), nor by objects from $B_{1}$($c_{67},c_{53},c_{35},c_{17})$, the procedure proceeded to Step G6.
Step G6: Object $c_{54}$ was presented to the decision-maker: $f_{1}^{1}\left(c_{54}\right)=16$, $f_{1}^{2}\left(c_{54}\right)=77$, $f_{1}^{3}\left(c_{54}\right)=100$.
Step G7: The decision-maker stated that, due to the insufficient value of the first criterion, $c_{54}$ could not be considered a good object.
Step G8: $c_{54}$ was added to $R_{1}$ and removed from ${\tilde{C}}_{1}$: $R_{1}∶=R_{1}\cup \left\{c_{54}\right\}$, ${\tilde{C}}_{1}∶={\tilde{C}}_{1}\backslash \left\{c_{54}\right\}$; the procedure proceeded to Step G3.
Step G3: The object that achieved the highest score for the second criterion was $c_{84}$.
Step G4: As $R_{1}\neq \emptyset \;and\;G_{1}\neq \emptyset$, the procedure proceeded to Step G5.
Step G5: As $c_{84}$ was neither dominated by objects from $G_{1}$ ($c_{62},c_{6}$), nor by objects from $B_{1}$($c_{67},c_{53},c_{35},c_{17},c_{54})$, the procedure proceeded to Step G6.
Step G6: Object $c_{84}$ was presented to the decision-maker: $f_{1}^{1}\left(c_{84}\right)=67$, $f_{1}^{2}\left(c_{84}\right)=76$, $f_{1}^{3}\left(c_{84}\right)=98$.
Step G7: The decision-maker accepted $c_{84}$ as a good object; the procedure proceeded to Step G9.
Step G9: $c_{84}$ was added to $G_{1}$ and removed from ${\tilde{C}}_{1}$: $G_{1}∶=G_{1}\cup \left\{c_{84}\right\}$, ${\tilde{C}}_{1}∶={\tilde{C}}_{1}\backslash \left\{c_{84}\right\}$, $m_{1}^{G}=3$.
Step G10: As $m_{1}^{G}=n_{1}^{G}$, the identification of good objects was completed.

Identification of the stage set of bad objects for stage *t* = 1:

Step B1: ${\tilde{C}}_{1}∶=C_{1}\backslash \left\{c_{62},c_{6},c_{84}\right\}$, $R_{1}≔\emptyset ,s=1$.
Step B2: As $h_{1}^{i}=\;1$ for i=1, l=1.
Step B3: The object that achieved the lowest score for the first criterion was $c_{58}$.
Step B4: As $R_{1}=\emptyset$ and $B_{1}=\emptyset$, the procedure proceeded to Step B6.
Step B6: Object $c_{58}$ was presented to the decision-maker: $f_{1}^{1}\left(c_{58}\right)=1$, $f_{1}^{2}\left(c_{58}\right)=79$ and $f_{1}^{3}\left(c_{58}\right)=11$.
Step B7: The decision-maker accepted $c_{58}$ as a bad object; the procedure proceeded to Step B9.
Step B9: $c_{58}$ was added to $B_{1}$ and removed from ${\tilde{C}}_{1}$: $B_{1}∶=B_{1}\cup \left\{c_{58}\right\}$, ${\tilde{C}}_{1}∶={\tilde{C}}_{1}\backslash \left\{c_{58}\right\}$, $m_{1}^{B}=1$.
Step B10: As $m_{1}^{B}<n_{1}^{B}$ and ${\tilde{C}}_{1}\neq \emptyset$, the identification of bad objects was continued.
Step B11: s∶=2; the procedure proceeded to Step B2.
Step B2: As $h_{1}^{i}=\;2$ for i=2, l=2.
Step B3: The object that achieved the lowest score for the second criterion was $c_{55}$.
Step B4: As $B_{1}\neq \emptyset$, the procedure proceeded to Step B5.
Step B5: As $c_{55}$ did not dominate $c_{58}$ (the only object in $B_{1}$), the procedure proceeded to Step B6.
Step B6: Object $c_{55}$ was presented to the decision-maker: $f_{1}^{1}\left(c_{55}\right)=44$, $f_{1}^{2}\left(c_{55}\right)=1$, $f_{1}^{3}\left(c_{55}\right)=23$.
Step B7: The decision-maker accepted $c_{55}$ as a bad object; the procedure proceeded to Step B9.
Step B9: $c_{55}$ was added to $B_{1}$ and removed from ${\tilde{C}}_{1}$: $B_{1}∶=B_{1}\cup \left\{c_{55}\right\}$, ${\tilde{C}}_{1}∶={\tilde{C}}_{1}\backslash \left\{c_{55}\right\}$, $m_{1}^{B}=2$.
Step B10: As $m_{1}^{B}<n_{1}^{B}$ and ${\tilde{C}}_{1}\neq \emptyset$, the identification of bad objects was continued.
Step B11: s∶=3; the procedure proceeded to Step B2.
Step B2: As $h_{1}^{i}=\;3$ for i=3, l=3.
Step B3: The object that achieved the lowest score for the third criterion was $c_{35}$ (which had been previously considered as a candidate for a good object).
Step B4: As $B_{1}\neq \emptyset$ procedure proceeded to Step B5.
Step B5: As $c_{35}$ did not dominate any of the objects from $B_{1}$ ($c_{58},c_{55}$), the procedure proceeded to Step B6.
Step B6: Object $c_{54}$ was presented to the DM: $f_{1}^{1}\left(c_{35}\right)=34$, $f_{1}^{2}\left(c_{35}\right)=98$, $f_{1}^{3}\left(c_{35}\right)=1$.
Step B7: The decision-maker stated that, due to the high value of the second criterion, $c_{35}$ could not be considered a bad object.
Step B8: $c_{35}$ was added to $R_{1}$ and removed from ${\tilde{C}}_{1}$: $R_{1}∶=R_{1}\cup \left\{c_{35}\right\}$, ${\tilde{C}}_{1}∶={\tilde{C}}_{1}\backslash \left\{c_{35}\right\}$; the procedure proceeded to Step B3.
Step B3: There were three objects in ${\tilde{C}}_{1}$ that achieved the lowest value of the third criterion, equal to 2: $c_{2},c_{69}$ and $c_{90}$; taking into account the hierarchy of criteria, $c_{90}$ was selected as a candidate for a good object.
Step B4: As $B_{1}\neq \emptyset$, the procedure proceeded to Step B5.
Step B5: As $c_{90}$ did not dominate any of the objects from $B_{1}$ ($c_{58},c_{55}$), nor the object $c_{35}$ (the only object from $R_{1}$), the procedure proceeded to Step B6.
Step B6: Object $c_{90}$ was presented to the DM: $f_{1}^{1}\left(c_{90}\right)=6$, $f_{1}^{2}\left(c_{90}\right)=73$, $f_{1}^{3}\left(c_{90}\right)=2$.
Step B7: The decision-maker accepted $c_{90}$ as a bad object; the procedure proceeded to Step B9.
Step B9: $c_{90}$ was added to $B_{1}$ and removed from ${\tilde{C}}_{1}$: $B_{1}∶=B_{1}\cup \left\{c_{90}\right\}$, ${\tilde{C}}_{1}∶={\tilde{C}}_{1}\backslash \left\{c_{90}\right\}$, $m_{1}^{B}=3$.
Step B10: As $m_{1}^{B}=n_{1}^{B}$, the identification of bad objects was complete.

The sets of good and bad objects for stage 1 are presented in Table 1. The same procedure was applied for identifying good and bad objects for stages 2 and 3. At each of these stages, the decision-maker set the number of good and bad objects at three. In stage two, the hierarchy of criteria was established as follows: the most important criterion was No. 2, followed by criterion No. 3, and the least important was criterion No. 1. In stage 3, the hierarchy was established as follows: criterion 3, criterion 1, criterion 2. The calculations were similar to those for stage 1: first, the object with the highest rating for the most important criterion was proposed, followed by the objects with the highest ratings for the subsequent criteria in accordance with the established hierarchy. The results are presented in Table 2 and Table 3. In these tables, objects are denoted both according to the notation used above $\left(c_{i}\right)$ and according to the notation used below in Example 2 $\left(g_{t}^{\left(\cdot \right)},b_{t}^{\left(\cdot \right)}\right)$.

## 5. Application in Resource Allocation in Spatial Development Planning Problem

### 5.1. Assumptions and Numerical Data

Three synthetic measures will be used, which play the roles of criteria in the Multistage Bipolar method:

- A measure of economic development (criterion 1);
- A measure of social development (criterion 2);
- A measure of environmental development (criterion 3).

We assume that each of them can take values on a scale from 0 to 100. We want to achieve the sustainable development goals set by the decision-maker as best as possible.

We will consider this issue as a multistage decision process, taking into account a time horizon consisting of short, medium and long periods. Our considerations will be based on the Multistage Bipolar method, in which we will use the stage “good” and “bad” objects generated in Example 1 by means of the interactive procedure proposed in this paper. These sets are shown for subsequent stages in Table 1, Table 2 and Table 3, respectively.

In Stage 1, the decision-maker places a particular emphasis on economic development; in Stage 2, on social development and in Stage 3, on environmental development. This is reflected in the adopted set of weights, presented in Table 4.

The achievable values of the synthetic measures considered depend on the amount of expenditure incurred. Four levels of expenditure, related to the orders considered, were estimated, a very low level, low level, medium level and high level, to which we assigned the numbers 0, 1, 2 and 3. The numerical values that we considered further are shown in Table 5.

We assume that at the first stage, the total level of expenditures will be equal to 3. We can state 10 stage alternatives in stage 1: $a_{1}^{\left(1\right)}$, …, $a_{1}^{\left(10\right)}$. The stage alternative $a_{1}^{\left(1\right)}$ has the form $a_{1}^{\left(1\right)}=(3,0,0)$. It means that the level of expenditures for economic order is equal to 3 (high expenditure level), the level of expenditure for social development is equal to 0 (very low expenditure level) and the level of expenditures for environmental development is equal to 0 (very low expenditure level). From Table 5, we find that for this stage alternatives, the values of the synthetic measures are as follows:

$$f_{1}^{1}\left(a_{1}^{\left(1\right)}\right)=70,\;f_{1}^{2}\left(a_{1}^{\left(1\right)}\right)=12,\;f_{1}^{3}\left(a_{1}^{\left(1\right)}\right)=8.$$

In a similar way, we interpret the stage alternatives, $a_{1}^{\left(2\right)}$, …, $a_{1}^{\left(10\right)}$. All these stage alternatives are shown in Table A4 (see Appendix B).

We assume that, in the second stage, the total level of expenditures will be equal to 4. We can state 12 stage alternatives: $a_{2}^{\left(1\right)}$, …, $a_{2}^{\left(12\right)}$. The stage alternative $a_{2}^{\left(1\right)}$ has the form $a_{2}^{\left(1\right)}=(3,\;1,\;0)$. From Table 5 we find that, for this stage alternative, the values of the synthetic measures are as follows:

$$f_{2}^{1}\left(a_{2}^{\left(1\right)}\right)=70,\;f_{2}^{2}\left(a_{2}^{\left(1\right)}\right)=20,\;f_{2}^{3}\left(a_{2}^{\left(1\right)}\right)=8$$

In a similar way, we interpret the stage alternatives $a_{2}^{\left(2\right)}$, …, $a_{2}^{\left(12\right)}$. All these stage alternatives are shown in Table A5 (see Appendix B).

We assume that in the third stage, the total level of expenditures will be equal to 5. We can state 12 stage alternatives: $a_{3}^{\left(1\right)}$, …, $a_{3}^{\left(12\right)}$. The stage alternative $a_{3}^{\left(1\right)}$ has the form $a_{3}^{\left(1\right)}=(3,\;2,\;0)$. From Table 5 we find that for this stage alternative, the values of the synthetic measures are as follows:

$$f_{3}^{1}\left(a_{3}^{\left(1\right)}\right)=70,\;f_{3}^{2}\left(a_{3}^{\left(1\right)}\right)=20,\;f_{3}^{3}\left(a_{3}^{\left(1\right)}\right)=8$$

In a similar way, we interpret the stage alternatives $a_{3}^{\left(2\right)}$, …, $a_{3}^{\left(12\right)}$. All these stage alternatives are shown in Table A6 (see Appendix B).

In Example 2, each multistage alternative is defined as follows:

$$a=(a_{1,\;}a_{2,\;}a_{3})$$

(see Formula 1). As

$$card\;A_{1}=10,\;\;\;\;\;card\;A_{2}=12,\;\;\;\;\;card\;A_{3}=12,$$

we have a total of 10 × 12 × 12 = 1440 multistage alternatives, which we number from 1 to 1440. We arrange them in lexicographic order, shown in Table A7 (see Appendix B).

For example, let us consider the multistage alternative $a^{\left(15\right)}=\left[a_{1}^{\left(1\right)},a_{2}^{\left(2\right)},a_{3}^{\left(1\right)}\right]$. This development scenario is characterized by the following:

- High expenditures in economic development in stage 1, medium expenditures in economic development in stage 2 and high expenditures in economic development in stage 3;
- Very low expenditures in social development in stage 1, medium expenditures in social development in stage 2 and medium expenditures in social development in stage 3;
- Very low expenditures in environmental development at stage 1, very low expenditures in environmental development in stage 2 and very low expenditures in environmental development in stage 3.

The decision-maker applied the interactive method described in the previous chapters of the paper and obtained (Example 1) three good objects and three bad objects at each stage. We have the following stage sets of good and bad objects:

$$G_{1}=\{g_{1}^{\left(1\right)},\;g_{1}^{\left(2\right)},g_{1}^{\left(3\right)}\}\;\;\;\;\;\;\;\;\;\;\;\;\;\;\;\;\;\;B_{1}=\left\{b_{1}^{\left(1\right)},\;b_{1}^{\left(2\right)},b_{1}^{\left(3\right)}\right\}$$

$$G_{2}=\{g_{2}^{\left(1\right)},\;g_{2}^{\left(2\right)},g_{2}^{\left(3\right)}\}\;\;\;\;\;\;\;\;\;\;\;\;\;\;\;\;\;\;B_{2}=\left\{b_{2}^{\left(1\right)},\;b_{2}^{\left(2\right)},b_{2}^{\left(3\right)}\right\}$$

$$G_{3}=\{g_{3}^{\left(1\right)},\;g_{3}^{\left(2\right)},g_{3}^{\left(3\right)}\}\;\;\;\;\;\;\;\;\;\;\;\;\;\;\;\;\;\;B_{3}=\left\{b_{3}^{\left(1\right)},\;b_{3}^{\left(2\right)},b_{3}^{\left(3\right)}\right\}$$

The values of the stage criteria are presented in Table 1, Table 2 and Table 3, respectively.

### 5.2. Using the Multistage Bipolar Method to Select the Best Multistage Scenario

Step 1: We compare the stage alternative $a_{t}$ with the stage reference objects $r_{t}$, where $r_{t}$ is taken as the objects $b_{t}$ and $g_{t}$, respectively. For each criterion *k* (*k* = 1, …, *K*), we calculate the differences:

(12) $${⸹}_{t}^{k}\left(a_{t},r_{t}\right)=\;f_{t}^{k}\left(a_{t}\right)-f_{t}^{k}\left(r_{t}\right)$$

Next we calculate values:

(13) $$S({⸹}_{t}^{k}(a_{t},r_{t}))=\{\begin{array}{cc} 0 & if\;\;\;\;{⸹}_{t}^{k}\left(a_{t},r_{t}\right)\leq 0\\ 1 & if\;\;\;{⸹}_{t}^{\left(k\right)}\left(a_{t},r_{t}\right)>0 \end{array}$$

If $S({⸹}_{t}^{k}(a_{t},r_{t}))=1,\;$it means that the stage alternative $a_{t}$ prevails over the stage reference object $r_{t}$ with respect to the *k*-th criterion.

Comparing $r_{t}$ with $a_{t}$ we apply the following formula:

(14) $${S(⸹}_{t}^{k}(r_{t},a_{t}))=1-S({⸹}_{t}^{k}(a_{t},r_{t}))$$

If ${S(⸹}_{t}^{k}(r_{t},a_{t}))=1,\;$it means that the stage reference object $r_{t}$ prevails over the stage alternative $a_{t}$ with respect to the *k*-th criterion.

Using stage weights from Table 4, we calculate the following:

(15) $$\Psi_{t}\left(a_{t},r_{t}\right)=\sum_{k=1}^{K}w_{t}^{k}{S(⸹}_{t}^{k}(a_{t},r_{t}))$$

According to Equation (14), the following relationship occurs:

(16) $${\Psi_{t}\left(r_{t},a_{t}\right)=1-\Psi }_{t}(a_{t},r_{t})$$

The value of $\Psi_{t}(a_{t}$,$r_{t}$) determines the degree of advantage of $a_{t}$ over $r_{t}$, while $\Psi_{t}(r_{t}$,$a_{t}$) determines the degree of advantage of $r_{t}$ over $a_{t}$. The calculated values of the functions $\Psi_{t}(a_{t}$,$b_{t}$) and $\Psi_{t}(a_{t}$,$g_{t}$) for t = 1, 2, 3 are presented in Table A8 (see Appendix B).

Step 2: To compare the stage alternatives with the set of stage reference bad objects, we calculate the flow values:

- Positive:

(17) $$\Phi_{R_{t}}^{+}\left(a_{t}\right)=\frac{1}{r_{t}}\sum_{r_{t}\in R_{t}}\Psi (a_{t},r_{t})$$

- Negative:

(18) $$\Phi_{R_{t}}^{-}\left(a_{t}\right)=\frac{1}{r_{t}}\sum_{r_{t}\in R_{t}}\Psi (r_{t},a_{t})$$

- Net:

(19) $$\Phi_{R_{t}}\left(a_{t}\right)=\Phi_{R_{t}}^{+}\left(a_{t}\right)-\Phi_{R_{t}}^{-}\left(a_{t}\right)$$

where $r_{t}$ denotes the cardinality of the corresponding reference set $R_{t}$. The calculation results for net flows are presented in Table A9 (see Appendix B).

Step 3: We determine the position of multistage alternatives by calculating the following for each multistage alternative:

- The multistage failure avoidance degree:

(20) $$\Phi_{B}\left(a\right)=\frac{1}{T}\sum_{t=1}^{T}\Phi_{B_{t}}\left(a_{t}\right)$$

- The multistage success achievement degree:

(21) $$\Phi_{G}\left(a\right)=\frac{1}{T}\sum_{t=1}^{T}\Phi_{G_{t}}\left(a_{t}\right)$$

Step 4: We sort multistage alternatives $a^{\left(i\right)}$, assigning them to predefined classes:

(22) $$A^{H}=\left\{a\in A:\Phi_{B}\left(a\right)\geq 0,\Phi_{G}\left(a\right)\geq 0\right\}$$

(23) $$A^{M}=\left\{a\in A:\Phi_{B}\left(a\right)\geq 0,\Phi_{G}\left(a\right)<0\right\},$$

(24) $$A^{L}=\left\{a\in A:\Phi_{B}\left(a\right)<0,\Phi_{G}\left(a\right)<0\right\}.$$

Using the calculated values $\Phi_{B}\left(a^{\left(i\right)}\right)$, $\Phi_{G}\left(a^{\left(i\right)}\right)$, we state that set **A***^H^* is empty and all the multistage alternatives belong to classes **A***^M^* or **A***^L^*.

Step 5: Ranking multistage alternatives. To find the best multistage alternative, we calculate the values *d*($a^{\left(i\right)}$):

(25) $$d\left(a\right)=\Phi_{G}\left(a\right)+\Phi_{B}\left(a\right)$$

for consecutive multistage alternatives from class $A^{M}.\;$The 20 best-rated multistage alternatives are shown in Table 6.

### 5.3. Analysis of Numerical Results

When presenting the issue of planning the distribution of funds for sustainable development of the region, 1440 scenarios were considered. This number is the result of combining successive stage scenarios from earlier stages with all possible stage scenarios in later stages. The set of the best alternatives consists of five elements. They are the following multistage alternatives:

$$a^{\left(13\right)}=\;[a_{1}^{\left(1\right)},\;a_{2}^{\left(2\right)},\;a_{3}^{\left(1\right)}]=[\left(3,\;0,\;0\right),\;\left(2,\;2,\;0\right),\;\left(3,\;2,\;0\right)],$$

$$a^{\left(25\right)}=[a_{1}^{\left(1\right)},a_{2}^{\left(3\right)},\;\;a_{3}^{\left(1\right)}]=\left[\left(3,\;0,\;0\right),\;\left(1,\;3,\;0\right),\;\left(3,\;2,\;0\right)\right],$$

$$a^{\left(61\right)}=[a_{1}^{\left(1\right)},a_{2}^{\left(6\right)},\;a_{3}^{\left(1\right)}]=\left[\left(3,\;0,\;0\right),\;\left(1,\;2,\;1\right),\;\left(3,\;2,\;0\right)\right],$$

$$a^{\left(73\right)}=[a_{1}^{\left(1\right)},a_{2}^{\left(7\right)},a_{3}^{\left(1\right)}]=[\left(3,\;0,\;0\right),\;\left(0,\;3,\;1\right),\;\left(3,\;2,\;0\right)],$$

$$a^{\left(109\right)}=[a_{1}^{\left(1\right)},a_{2}^{\left(10\right)},\;a_{3}^{\left(1\right)}]=[\left(3,\;0,\;0\right),\;\left(0,\;2,\;2\right),\;\left(3,\;2,\;0\right)].$$

In all the best solutions, expenditures on economic development are preferred. The most balanced multistage alternative appears to be multistage alternative $a^{\left(109\right)}$. This development scenario is characterized by the following:

- High expenditures in economic development in stage 1, very low expenditures in economic development in stage 2 and high expenditures in economic development in stage 3;
- Very low expenditures in social development in stage 1, medium expenditures in social development in stage 2 and medium expenditures in social development in stage 3;
- Very low expenditures in environmental development at stage 1, medium expenditure development in stage 2 and very low expenditures in environmental development in stage 3.

The calculations presented in this paper were performed using an Excel spreadsheet, a single set of weights and a single set of stage reference sets were utilized. It is expected that modifying the values of these weights and preferences regarding the development sequence would yield different development scenarios, which should be subjected to further analysis. It would be interesting to investigate the sensitivity of the solution to changing weights and sets of reference objects, which would require the development of specialized software.

## 6. Conclusions

This paper presents a synthesis of three streams related to multi-criteria decision-making: the reference point-based approach, the interactive approach and multistage decision processes. Particular attention is devoted to the interactive selection of objects forming the stage reference sets. The proposed procedure represents a first proposal for solving this problem and may be modified depending on decision-makers’ opinions.

The procedure is based on an interactive approach. This approach inherently results in both some advantages and limitations in its application. Its primary advantage is its simplicity.

In each iteration, the decision-maker is presented with a specific object, which they can either accept as “good” or “bad,” or indicate why it cannot be classified as such. In the latter case, the information provided by the decision-maker is subsequently used to determine further proposals. Consequently, the decision-maker does not have to provide all the information necessary to determine a solution at once, but can articulate it step by step. By monitoring the results, the decision-maker expands their knowledge regarding the essence of the problem being solved.

However, the use of this type of approach has its limitations. It may happen that the decision-maker’s expectations regarding the objects to be considered “good” or “bad” are too high, resulting in a failure to obtain the assumed number of reference objects. In such a case, it would be necessary to restart the procedure, which could, of course, lower the decision-maker’s confidence in the obtained results.

It should also be remembered that the fundamental assumption of the procedure is the availability of sets of candidates from which “good” or “bad” objects are selected. In the presented example, this set was generated randomly, based on knowledge of the value ranges within which the analyzed criteria fall. However, objects generated in this manner may be perceived by the decision-maker as differing too much from the alternatives they might encounter in reality. If the problem is repetitive—meaning it has been solved previously for a different data set—a better solution may be to utilize historical data.

The problem addressed constitutes a mathematical economics model, while simultaneously demonstrating that multi-criteria methods are widely applicable in management. When analyzing the specific nature of managerial decision-making problems, attention is drawn to their multidimensional character. Typically, they require evaluation based on multiple criteria of both a quantitative and qualitative nature.

This situation is also encountered in public management. In striving to effectively fulfill their tasks, public administration units must employ methods proven in the private sector. Of fundamental importance here is the expenditure of public funds in a manner that yields maximum benefits for citizens.

## Figures and Tables

**Table 1:** The sets of good and bad objects for stage 1.

Good Objects				Bad Objects			
Object	$f_{1}^{1}\left(\cdot \right)$	$f_{1}^{2}\left(\cdot \right)$	$f_{1}^{3}\left(\cdot \right)$	Object	$f_{1}^{1}\left(\cdot \right)$	$f_{1}^{2}\left(\cdot \right)$	$f_{1}^{3}\left(\cdot \right)$
$c_{62}\equiv g_{1}^{\left(1\right)}$	99	85	52	$c_{58}\equiv b_{1}^{\left(1\right)}$	1	79	11
$c_{6}\equiv g_{1}^{\left(2\right)}$	85	95	13	$c_{55}\equiv b_{1}^{\left(2\right)}$	44	1	23
$c_{84}\equiv g_{1}^{\left(3\right)}$	67	76	98	$c_{90}\equiv b_{1}^{\left(3\right)}$	6	73	2

**Table 2:** The sets of good and bad objects for stage 2.

Good Objects				Bad Objects			
Object	$f_{2}^{1}\left(\cdot \right)$	$f_{2}^{2}\left(\cdot \right)$	$f_{2}^{3}\left(\cdot \right)$	Object	$f_{2}^{1}\left(\cdot \right)$	$f_{2}^{2}\left(\cdot \right)$	$f_{2}^{3}\left(\cdot \right)$
$c_{10}\equiv g_{2}^{\left(1\right)}$	66	99	92	$c_{88}\equiv b_{2}^{\left(1\right)}$	51	2	35
$c_{86}\equiv g_{2}^{\left(2\right)}$	64	40	96	$c_{18}\equiv b_{2}^{\left(2\right)}$	52	28	1
$c_{70}\equiv g_{2}^{\left(3\right)}$	99	76	57	$c_{100}\equiv b_{2}^{\left(3\right)}$	1	23	34

**Table 3:** The sets of good and bad objects for stage 3.

Good Objects				Bad Objects			
Object	$f_{3}^{1}\left(\cdot \right)$	$f_{3}^{2}\left(\cdot \right)$	$f_{3}^{3}\left(\cdot \right)$	Object	$f_{3}^{1}\left(\cdot \right)$	$f_{3}^{2}\left(\cdot \right)$	$f_{3}^{3}\left(\cdot \right)$
$c_{23}\equiv g_{3}^{\left(1\right)}$	76	38	100	$c_{48}\equiv b_{3}^{\left(1\right)}$	8	78	4
$c_{10}\equiv g_{3}^{\left(2\right)}$	96	13	79	$c_{30}\equiv b_{3}^{\left(2\right)}$	1	28	67
$c_{79}\equiv g_{3}^{\left(3\right)}$	36	98	69	$c_{6}\equiv b_{3}^{\left(3\right)}$	63	2	25

**Table 4:** Criterion weights.

	Weights		
Stage	Criterion 1	Criterion 2	Criterion 3
1	0.45	0.35	0.20
2	0.20	0.45	0.35
3	0.35	0.20	0.45

**Table 5:** Expenditures and values of synthetic measures.

		Values of Synthetic Measures		
Level	Expenditures	Economic Development	Social Development	Environmental Development
0	Very low expenditure level	8	12	10
1	Low expenditure level	18	20	21
2	Medium expenditure level	43	42	45
3	High expenditure level	70	66	71

**Table 6:** Ranking multistage alternatives.

No.	a	$a_{1}$	$a_{2}$	$a_{3}$	$\Phi_{B}\left(a\right)$	$\Phi_{G}\left(a\right)$	*d*(a)
1	$a^{\left(13\right)}$	$a_{1}^{\left(1\right)}$	$a_{2}^{\left(2\right)}$	$a_{3}^{\left(1\right)}$	−0.6333	0.0889	−0.5444
2	$a^{\left(25\right)}$	$a_{1}^{\left(1\right)}$	$a_{2}^{\left(3\right)}$	$a_{3}^{\left(1\right)}$	−0.6333	0.0889	−0.5444
3	$a^{\left(61\right)}$	$a_{1}^{\left(1\right)}$	$a_{2}^{\left(6\right)}$	$a_{3}^{\left(1\right)}$	−0.6333	0.0889	−0.5444
4	$a^{\left(73\right)}$	$a_{1}^{\left(1\right)}$	$a_{2}^{\left(7\right)}$	$a_{3}^{\left(1\right)}$	−0.6333	0.0889	−0.5444
5	$a^{\left(109\right)}$	$a_{1}^{\left(1\right)}$	$a_{2}^{\left(10\right)}$	$a_{3}^{\left(1\right)}$	−0.6333	0.0889	−0.5444
6	$a^{\left(1021\right)}$	$a_{1}^{\left(8\right)}$	$a_{2}^{\left(2\right)}$	$a_{3}^{\left(1\right)}$	−0.6889	0.0778	−0.6111
7	$a^{\left(1033\right)}$	$a_{1}^{\left(8\right)}$	$a_{2}^{\left(3\right)}$	$a_{3}^{\left(1\right)}$	−0.6889	0.0778	−0.6111
8	$a^{\left(1069\right)}$	$a_{1}^{\left(8\right)}$	$a_{2}^{\left(6\right)}$	$a_{3}^{\left(1\right)}$	−0.6889	0.0778	−0.6111
9	$a^{\left(1081\right)}$	$a_{1}^{\left(8\right)}$	$a_{2}^{\left(7\right)}$	$a_{3}^{\left(1\right)}$	−0.6889	0.0778	−0.6111
10	$a^{\left(1117\right)}$	$a_{1}^{\left(8\right)}$	$a_{2}^{\left(10\right)}$	$a_{3}^{\left(1\right)}$	−0.6889	0.0778	−0.6111
11	$a^{\left(1165\right)}$	$a_{1}^{\left(9\right)}$	$a_{2}^{\left(2\right)}$	$a_{3}^{\left(1\right)}$	−0.6889	0.0778	−0.6111
12	$a^{\left(1177\right)}$	$a_{1}^{\left(9\right)}$	$a_{2}^{\left(3\right)}$	$a_{3}^{\left(1\right)}$	−0.6889	0.0778	−0.6111
13	$a^{\left(1213\right)}$	$a_{1}^{\left(9\right)}$	$a_{2}^{\left(6\right)}$	$a_{3}^{\left(1\right)}$	−0.6889	0.0778	−0.6111
14	$a^{\left(1225\right)}$	$a_{1}^{\left(9\right)}$	$a_{2}^{\left(7\right)}$	$a_{3}^{\left(1\right)}$	−0.6889	0.0778	−0.6111
15	$a^{\left(1261\right)}$	$a_{1}^{\left(9\right)}$	$a_{2}^{\left(10\right)}$	$a_{3}^{\left(1\right)}$	−0.6889	0.0778	−0.6111
16	$a^{\left(1309\right)}$	$a_{1}^{\left(10\right)}$	$a_{2}^{\left(2\right)}$	$a_{3}^{\left(1\right)}$	−0.6889	0.0778	−0.6111
17	$a^{\left(1321\right)}$	$a_{1}^{\left(10\right)}$	$a_{2}^{\left(3\right)}$	$a_{3}^{\left(1\right)}$	−0.6889	0.0778	−0.6111
18	$a^{1357}$	$a_{1}^{\left(10\right)}$	$a_{2}^{\left(6\right)}$	$a_{3}^{\left(1\right)}$	−0.6889	0.0778	−0.6111
19	$a^{\left(1369\right)}$	$a_{1}^{\left(10\right)}$	$a_{2}^{\left(7\right)}$	$a_{3}^{\left(1\right)}$	−0.6889	0.0778	−0.6111
20	$a^{\left(1405\right)}$	$a_{1}^{\left(10\right)}$	$a_{2}^{\left(10\right)}$	$a_{3}^{\left(1\right)}$	−0.6889	0.0778	−0.6111

## Data Availability

The original contributions presented in this study are included in the article. Further inquiries can be directed to the corresponding author.

## Appendix A

Table A1, Table A2 and Table A3 present sets of candidates for good and bad objects generated randomly for stages 1, 2 and 3, respectively.

**Table A1:** The set of candidates for good and bad objects generated randomly for stage 1.

No.	$\left(f_{1}^{1}\left(\cdot \right);f_{1}^{2}\left(\cdot \right);f_{1}^{3}\left(\cdot \right)\right)$	No.	$\left(f_{1}^{1}\left(\cdot \right);f_{1}^{2}\left(\cdot \right);f_{1}^{3}\left(\cdot \right)\right)$	No.	$\left(f_{1}^{1}\left(\cdot \right);f_{1}^{2}\left(\cdot \right);f_{1}^{3}\left(\cdot \right)\right)$	No.	$\left(f_{1}^{1}\left(\cdot \right);f_{1}^{2}\left(\cdot \right);f_{1}^{3}\left(\cdot \right)\right)$
1	(75; 20; 91)	26	(30; 65; 52)	51	(26; 71; 42)	76	(2; 44; 69)
2	(98; 43; 2)	27	(73; 33; 67)	52	(51; 7; 39)	77	(24; 92; 67)
3	(10; 27; 76)	28	(86; 57; 23)	53	(38; 100; 5)	78	(31; 91; 96)
4	(27; 43; 54)	29	(10; 77; 56)	54	(16; 77; 100)	79	(89; 67; 3)
5	(89; 92; 76)	30	(39; 24; 53)	55	(44; 1; 23)	80	(81; 30; 10)
6	(85; 95; 13)	31	(64; 70; 91)	56	(36; 14; 61)	81	(40; 74; 26)
7	(76; 44; 56)	32	(52; 38; 73)	57	(97; 25; 31)	82	(39; 23; 87)
8	(2; 94; 31)	33	(90; 86; 36)	58	(1; 79; 11)	83	(49; 21; 50)
9	(86; 70; 89)	34	(29; 29; 95)	59	(74; 79; 60)	84	(67; 76; 98)
10	(59; 66; 28)	35	(34; 98; 1)	60	(61; 15; 77)	85	(99; 37; 35)
11	(80; 52; 66)	36	(40; 33; 6)	61	(11; 21; 47)	86	(67; 82; 22)
12	(33; 38; 69)	37	(18; 57; 43)	62	(99; 85; 52)	87	(13; 69; 20)
13	(34; 94; 14)	38	(46; 77; 66)	63	(54; 88; 73)	88	(96; 87; 75)
14	(80; 18; 83)	39	(44; 77; 26)	64	(81; 91; 49)	89	(75; 27; 10)
15	(80; 20; 90)	40	(11; 28; 94)	65	(64; 84; 97)	90	(6; 73; 2)
16	(16; 60; 48)	41	(17; 51; 13)	66	(43; 79; 86)	91	(88; 75; 76)
17	(5; 98; 4)	42	(91; 29; 18)	67	(100; 10; 59)	92	(72; 19; 95)
18	(58; 70; 54)	43	(49; 53; 35)	68	(98; 22; 73)	93	(12; 71; 46)
19	(37; 9; 75)	44	(62; 34; 21)	69	(89; 80; 2)	94	(71; 70; 63)
20	(59; 21; 15)	45	(80; 94; 58)	70	(37; 18; 85)	95	(93; 4; 40)
21	(90; 38; 7)	46	(79; 91; 53)	71	(36; 51; 48)	96	(19; 59; 40)
22	(14; 91; 18)	47	(15; 61; 92)	72	(94; 2; 84)	97	(20; 38; 71)
23	(79; 19; 27)	48	(40; 20; 5)	73	(85; 15; 22)	98	(71; 86; 7)
24	(97; 87; 35)	49	(73; 22; 11)	74	(12; 47; 67)	99	(74; 89; 32)
25	(15; 64; 64)	50	(98; 14; 82)	75	(96; 27; 13)	100	(2; 8; 92)

**Table A2:** The set of candidates for good and bad objects generated randomly for stage 2.

No.	$\left(f_{2}^{1}\left(\cdot \right);f_{2}^{2}\left(\cdot \right);f_{2}^{3}\left(\cdot \right)\right)$	No.	$\left(f_{2}^{1}\left(\cdot \right);f_{2}^{2}\left(\cdot \right);f_{2}^{3}\left(\cdot \right)\right)$	No.	$\left(f_{2}^{1}\left(\cdot \right);f_{2}^{2}\left(\cdot \right);f_{2}^{3}\left(\cdot \right)\right)$	No.	$\left(f_{2}^{1}\left(\cdot \right);f_{2}^{2}\left(\cdot \right);f_{2}^{3}\left(\cdot \right)\right)$
1	(75; 40; 95)	26	(82; 54; 89)	51	(79; 15; 32)	76	(97; 14; 58)
2	(14; 17; 73)	27	(17; 13; 100)	52	(89; 20; 52)	77	(67; 18; 72)
3	(3; 45; 57)	28	(51; 78; 74)	53	(49; 80; 69)	78	(86; 16; 15)
4	(87; 87; 59)	29	(84; 3; 51)	54	(43; 91; 34)	79	(13; 58; 82)
5	(51; 45; 19)	30	(34; 5; 84)	55	(100; 78; 5)	80	(66; 26; 84)
6	(65; 24; 56)	31	(61; 19; 23)	56	(34; 6; 74)	81	(21; 33; 19)
7	(32; 68; 3)	32	(15; 95; 20)	57	(36; 18; 56)	82	(80; 87; 91)
8	(24; 4; 76)	33	(69; 28; 58)	58	(36; 84; 52)	83	(55; 51; 17)
9	(53; 77; 78)	34	(84; 18; 81)	59	(58; 28; 48)	84	(85; 57; 87)
10	(66; 99; 92)	35	(68; 12; 63)	60	(29; 11; 25)	85	(61; 22; 57)
11	(56; 42; 62)	36	(81; 86; 1)	61	(80; 9; 25)	86	(64; 40; 96)
12	(79; 96; 91)	37	(59; 96; 95)	62	(96; 33; 8)	87	(83; 65; 35)
13	(4; 60; 22)	38	(67; 66; 14)	63	(49; 68; 45)	88	(51; 2; 35)
14	(83; 69; 26)	39	(65; 5; 76)	64	(47; 35; 21)	89	(28; 54; 22)
15	(4; 91; 10)	40	(56; 63; 41)	65	(18; 38; 29)	90	(64; 17; 25)
16	(99; 33; 44)	41	(2; 34; 48)	66	(42; 9; 79)	91	(4; 23; 48)
17	(46; 46; 74)	42	(94; 21; 36)	67	(49; 61; 43)	92	(27; 25; 8)
18	(52; 28; 1)	43	(67; 37; 83)	68	(89; 31; 34)	93	(98; 58; 64)
19	(50; 77; 86)	44	(97; 8; 96)	69	(34; 10; 34)	94	(12; 75; 46)
20	(46; 41; 28)	45	(84; 26; 55)	70	(99; 76; 57)	95	(75; 23; 98)
21	(64; 45; 29)	46	(85; 74; 74)	71	(83; 6; 21)	96	(6; 2; 86)
22	(59; 85; 46)	47	(8; 99; 57)	72	(26; 45; 63)	97	(81; 71; 37)
23	(66; 26; 1)	48	(57; 31; 91)	73	(37; 13; 44)	98	(17; 73; 78)
24	(15; 97; 50)	49	(89; 98; 56)	74	(6; 82; 84)	99	(92; 85; 87)
25	(10; 44; 92)	50	(77; 64; 73)	75	(31; 88; 14)	100	(1; 23; 34)

**Table A3:** The set of candidates for good and bad objects generated randomly for stage 3.

No.	$\left(f_{3}^{1}\left(\cdot \right);f_{3}^{2}\left(\cdot \right);f_{3}^{3}\left(\cdot \right)\right)$	No.	$\left(f_{3}^{1}\left(\cdot \right);f_{3}^{2}\left(\cdot \right);f_{3}^{3}\left(\cdot \right)\right)$	No.	$\left(f_{3}^{1}\left(\cdot \right);f_{3}^{2}\left(\cdot \right);f_{3}^{3}\left(\cdot \right)\right)$	No.	$\left(f_{3}^{1}\left(\cdot \right);f_{3}^{2}\left(\cdot \right);f_{3}^{3}\left(\cdot \right)\right)$
1	(96; 39; 37)	26	(49; 96; 23)	51	(37; 9; 37)	76	(7; 43; 46)
2	(82; 8; 58)	27	(60; 76; 62)	52	(20; 81; 82)	77	(90; 55; 67)
3	(88; 48; 42)	28	(49; 12; 92)	53	(78; 25; 63)	78	(85; 12; 14)
4	(35; 44; 22)	29	(20; 24; 44)	54	(78; 9; 4)	79	(36; 98; 69)
5	(13; 10; 94)	30	(1; 28; 67)	55	(38; 95; 82)	80	(6; 29; 90)
6	(63; 2; 25)	31	(62; 62; 91)	56	(76; 51; 93)	81	(15; 27; 42)
7	(93; 82; 44)	32	(78; 77; 70)	57	(47; 40; 97)	82	(20; 70; 43)
8	(96; 99; 4)	33	(38; 2; 43)	58	(38; 92; 51)	83	(62; 48; 99)
9	(28; 50; 66)	34	(71; 17; 36)	59	(36; 29; 23)	84	(36; 92; 71)
10	(96; 13; 79)	35	(30; 76; 30)	60	(89; 1; 87)	85	(61; 39; 5)
11	(20; 73; 22)	36	(55; 28; 80)	61	(63; 73; 42)	86	(25; 77; 78)
12	(40; 26; 36)	37	(23; 87; 82)	62	(87; 24; 56)	87	(14; 67; 63)
13	(84; 24; 52)	38	(53; 69; 46)	63	(82; 25; 4)	88	(21; 2; 61)
14	(40; 91; 28)	39	(21; 56; 73)	64	(2; 87; 42)	89	(11; 59; 97)
15	(79; 75; 57)	40	(56; 28; 95)	65	(92; 3; 44)	90	(64; 86; 6)
16	(32; 58; 21)	41	(99; 5; 54)	66	(83; 13; 96)	91	(37; 89; 16)
17	(76; 28; 23)	42	(68; 75; 54)	67	(68; 6; 22)	92	(83; 5; 73)
18	(68; 29; 63)	43	(60; 11; 98)	68	(80; 63; 55)	93	(25; 81; 27)
19	(33; 33; 67)	44	(75; 27; 39)	69	(16; 76; 21)	94	(68; 23; 39)
20	(52; 76; 11)	45	(28; 22; 58)	70	(22; 67; 71)	95	(50; 54; 15)
21	(75; 17; 84)	46	(19; 37; 48)	71	(34; 12; 69)	96	(57; 35; 65)
22	(45; 6; 49)	47	(84; 94; 15)	72	(59; 2; 73)	97	(68; 17; 30)
23	(76; 38; 100)	48	(8; 78; 4)	73	(37; 71; 97)	98	(44; 9; 66)
24	(39; 64; 76)	49	(94; 28; 98)	74	(30; 26; 37)	99	(69; 31; 71)
25	(79; 15; 66)	50	(27; 2; 68)	75	(63; 69; 97)	100	(20; 47; 64)

## Appendix B

Table A4, Table A5, Table A6, Table A7, Table A8 and Table A9 present the results obtained during the calculation procedure described in Example 2.

**Table A4:** Scenarios of development (stage alternatives) for stage 1.

	Expenditure Levels			$\mathrm{Values}\;\mathrm{of}\;\mathrm{Synthetic}\;\mathrm{Measures}\;f_{1}^{k}(\cdot )$		
Stage Alternatives Stage 1	Economic Order	Social Order	Environmental Order	Economic Development (*k* = 1)	Social Development (*k* = 2)	Environmental Development (*k* = 3)
$a_{1}^{\left(1\right)}$	3	0	0	70	12	10
$a_{1}^{\left(2\right)}$	2	1	0	43	20	10
$a_{1}^{\left(3\right)}$	1	2	0	18	42	10
$a_{1}^{\left(4\right)}$	0	3	0	8	66	10
$a_{1}^{\left(5\right)}$	2	0	1	43	12	21
$a_{1}^{\left(6\right)}$	1	1	1	18	20	21
$a_{1}^{\left(7\right)}$	0	2	1	8	42	21
$a_{1}^{\left(8\right)}$	0	1	2	8	20	45
$a_{1}^{\left(9\right)}$	1	0	2	18	12	45
$a_{1}^{\left(10\right)}$	0	0	3	8	12	71

**Table A5:** Scenarios of development (stage alternatives) for stage 2.

	Expenditure Levels			$\mathrm{Values}\;\mathrm{of}\;\mathrm{Synthetic}\;\mathrm{Measures}\;f_{2}^{k}(\cdot )$		
Stage Alternatives Stage 2	Economic Order	Social Order	Environmental Order	Economic Development (*k* = 1)	Social Development (*k* = 2)	Environmental Development (*k* = 3)
$a_{2}^{\left(1\right)}$	3	1	0	70	20	10
$a_{2}^{\left(2\right)}$	2	2	0	43	42	10
$a_{2}^{\left(3\right)}$	1	3	0	18	66	10
$a_{2}^{\left(4\right)}$	3	0	1	70	12	21
$a_{2}^{\left(5\right)}$	2	1	1	43	20	21
$a_{2}^{\left(6\right)}$	1	2	1	18	42	21
$a_{2}^{\left(7\right)}$	0	3	1	8	66	21
$a_{2}^{\left(8\right)}$	2	0	2	43	12	45
$a_{2}^{\left(9\right)}$	1	1	2	18	20	45
$a_{2}^{\left(10\right)}$	0	2	2	8	42	45
$a_{2}^{\left(11\right)}$	1	0	3	18	12	71
$a_{2}^{\left(12\right)}$	0	1	3	8	20	71

**Table A6:** Scenarios of development (stage alternatives) for stage 3.

	Expenditure Levels			$\mathrm{Values}\;\mathrm{of}\;\mathrm{Synthetic}\;\mathrm{Measures}\;f_{3}^{k}(\cdot )$		
Stage Alternatives Stage 3	Economic Order	Social Order	Environmental Order	Economic Development (*k* = 1)	Social Development (*k* = 2)	Environmental Development (*k* = 3)
$a_{3}^{\left(1\right)}$	3	2	0	70	42	10
$a_{3}^{\left(2\right)}$	2	3	0	43	66	10
$a_{3}^{\left(3\right)}$	3	1	1	70	20	21
$a_{3}^{\left(4\right)}$	2	2	1	43	42	21
$a_{3}^{\left(5\right)}$	1	3	1	18	66	21
$a_{3}^{\left(6\right)}$	3	0	2	70	12	45
$a_{3}^{\left(7\right)}$	2	1	2	43	20	45
$a_{3}^{\left(8\right)}$	1	2	2	18	42	45
$a_{3}^{\left(9\right)}$	0	3	2	8	66	45
$a_{3}^{\left(10\right)}$	2	0	3	43	12	71
$a_{3}^{\left(11\right)}$	1	1	3	18	20	71
$a_{3}^{\left(12\right)}$	0	2	3	8	42	71

**Table A7:** Multistage alternatives.

Number of Multistage Alternatives	Stage 1	Stage 2	Stage 3	Number of Multistage Alternatives	Stage 1	Stage 2	Stage3
$a^{\left(1\right)}$	$a_{1}^{\left(1\right)}$	$a_{2}^{\left(1\right)}$	$a_{3}^{\left(1\right)}$	…	…	…	…
$a^{\left(2\right)}$	$a_{1}^{\left(1\right)}$	$a_{2}^{\left(1\right)}$	$a_{3}^{\left(2\right)}$	$a^{\left(1426\right)}$	$a_{1}^{\left(9\right)}$	$a_{2}^{\left(11\right)}$	$a_{3}^{\left(10\right)}$
$a^{\left(3\right)}$	$a_{1}^{\left(1\right)}$	$a_{2}^{\left(1\right)}$	$a_{3}^{\left(3\right)}$	$a^{\left(1427\right)}$	$a_{1}^{\left(9\right)}$	$a_{2}^{\left(11\right)}$	$a_{3}^{\left(11\right)}$
$a^{\left(4\right)}$	$a_{1}^{\left(1\right)}$	$a_{2}^{\left(1\right)}$	$a_{3}^{\left(4\right)}$	$a^{\left(1428\right)}$	$a_{1}^{\left(9\right)}$	$a_{2}^{\left(11\right)}$	$a_{3}^{\left(12\right)}$
$a^{\left(5\right)}$	$a_{1}^{\left(1\right)}$	$a_{2}^{\left(1\right)}$	$a_{3}^{\left(5\right)}$	$a^{\left(1429\right)}$	$a_{1}^{\left(10\right)}$	$a_{2}^{\left(12\right)}$	$a_{3}^{\left(1\right)}$
$a^{\left(6\right)}$	$a_{1}^{\left(1\right)}$	$a_{2}^{\left(1\right)}$	$a_{3}^{\left(6\right)}$	$a^{\left(1430\right)}$	$a_{1}^{\left(10\right)}$	$a_{2}^{\left(12\right)}$	$a_{3}^{\left(2\right)}$
$a^{\left(7\right)}$	$a_{1}^{\left(1\right)}$	$a_{2}^{\left(1\right)}$	$a_{3}^{\left(7\right)}$	$a^{\left(1431\right)}$	$a_{1}^{\left(10\right)}$	$a_{2}^{\left(12\right)}$	$a_{3}^{\left(3\right)}$
$a^{\left(8\right)}$	$a_{1}^{\left(1\right)}$	$a_{2}^{\left(1\right)}$	$a_{3}^{\left(8\right)}$	$a^{\left(1432\right)}$	$a_{1}^{\left(10\right)}$	$a_{2}^{\left(12\right)}$	$a_{3}^{\left(4\right)}$
$a^{\left(9\right)}$	$a_{1}^{\left(1\right)}$	$a_{2}^{\left(1\right)}$	$a_{3}^{\left(9\right)}$	$a^{\left(1433\right)}$	$a_{1}^{\left(10\right)}$	$a_{2}^{\left(12\right)}$	$a_{3}^{\left(5\right)}$
$a^{\left(10\right)}$	$a_{1}^{\left(1\right)}$	$a_{2}^{\left(1\right)}$	$a_{3}^{\left(10\right)}$	$a^{\left(1434\right)}$	$a_{1}^{\left(10\right)}$	$a_{2}^{\left(12\right)}$	$a_{3}^{\left(6\right)}$
$a^{\left(11\right)}$	$a_{1}^{\left(1\right)}$	$a_{2}^{\left(1\right)}$	$a_{3}^{\left(11\right)}$	$a^{\left(1435\right)}$	$a_{1}^{\left(10\right)}$	$a_{2}^{\left(12\right)}$	$a_{3}^{\left(7\right)}$
$a^{\left(12\right)}$	$a_{1}^{\left(1\right)}$	$a_{2}^{\left(1\right)}$	$a_{3}^{\left(12\right)}$	$a^{\left(1436\right)}$	$a_{1}^{\left(10\right)}$	$a_{2}^{\left(12\right)}$	$a_{3}^{\left(8\right)}$
$a^{\left(13\right)}$	$a_{1}^{\left(1\right)}$	$a_{2}^{\left(2\right)}$	$a_{3}^{\left(1\right)}$	$a^{\left(1437\right)}$	$a_{1}^{\left(10\right)}$	$a_{2}^{\left(12\right)}$	$a_{3}^{\left(9\right)}$
$a^{\left(14\right)}$	$a_{1}^{\left(1\right)}$	$a_{2}^{\left(2\right)}$	$a_{3}^{\left(1\right)}$	$a^{\left(1438\right)}$	$a_{1}^{\left(10\right)}$	$a_{2}^{\left(12\right)}$	$a_{3}^{\left(10\right)}$
$a^{\left(15\right)}$	$a_{1}^{\left(1\right)}$	$a_{2}^{\left(2\right)}$	$a_{3}^{\left(1\right)}$	$a^{\left(1439\right)}$	$a_{1}^{\left(10\right)}$	$a_{2}^{\left(12\right)}$	$a_{3}^{\left(11\right)}$
…	…	…	…	$a^{\left(1440\right)}$	$a_{1}^{\left(10\right)}$	$a_{2}^{\left(12\right)}$	$a_{3}^{\left(12\right)}$

**Table A8:** Values $\Psi_{t}(a_{t}$,$b_{t}$) and $\Psi_{t}(a_{t}$,$g_{t}$) for *t* = 1, 2, 3.

**Stage 1**							
$\left(a_{1},b_{1}\right)$	$\Psi_{1}\left(\cdot \right)$	$\left(a_{1},g_{1}\right)$	$\Psi_{1}\left(\cdot \right)$	$\left(a_{1},b_{1}\right)$	$\Psi_{1}\left(\cdot \right)$	$\left(a_{1},g_{1}\right)$	$\Psi_{1}\left(\cdot \right)$
$\left(a_{1}^{\left(1\right)},b_{1}^{\left(1\right)}\right)$	0.45	$\left(a_{1}^{\left(1\right)},g_{1}^{\left(1\right)}\right)$	0	$\left(a_{1}^{\left(6\right)},b_{1}^{\left(1\right)}\right)$	0.65	$\left(a_{1}^{\left(6\right)},g_{1}^{\left(1\right)}\right)$	0
$\left(a_{1}^{\left(1\right)},b_{1}^{\left(2\right)}\right)$	0.8	$\left(a_{1}^{\left(1\right)},g_{1}^{\left(2\right)}\right)$	0	$\left(a_{1}^{\left(6\right)},b_{1}^{\left(2\right)}\right)$	0.35	$\left(a_{1}^{\left(6\right)},g_{1}^{\left(2\right)}\right)$	0.2
$\left(a_{1}^{\left(1\right)},b_{1}^{\left(3\right)}\right)$	0.65	$\left(a_{1}^{\left(1\right)},g_{1}^{\left(3\right)}\right)$	0.45	$\left(a_{1}^{\left(6\right)},b_{1}^{\left(3\right)}\right)$	0.65	$\left(a_{1}^{\left(6\right)},g_{1}^{\left(3\right)}\right)$	0
$\left(a_{1}^{\left(2\right)},b_{1}^{\left(1\right)}\right)$	0.45	$\left(a_{1}^{\left(2\right)},g_{1}^{\left(1\right)}\right)$	0	$\left(a_{1}^{\left(7\right)},b_{1}^{\left(1\right)}\right)$	0.65	$\left(a_{1}^{\left(7\right)},g_{1}^{\left(1\right)}\right)$	0
$\left(a_{1}^{\left(2\right)},b_{1}^{\left(2\right)}\right)$	0.35	$\left(a_{1}^{\left(2\right)},g_{1}^{\left(2\right)}\right)$	0	$\left(a_{1}^{\left(7\right)},b_{1}^{\left(2\right)}\right)$	0.35	$\left(a_{1}^{\left(7\right)},g_{1}^{\left(2\right)}\right)$	0.2
$\left(a_{1}^{\left(2\right)},b_{1}^{\left(3\right)}\right)$	0.65	$\left(a_{1}^{\left(2\right)},g_{1}^{\left(3\right)}\right)$	0	$\left(a_{1}^{\left(7\right)},b_{1}^{\left(3\right)}\right)$	0.65	$\left(a_{1}^{\left(7\right)},g_{1}^{\left(3\right)}\right)$	0
$\left(a_{1}^{\left(3\right)},b_{1}^{\left(1\right)}\right)$	0.45	$\left(a_{1}^{\left(3\right)},g_{1}^{\left(1\right)}\right)$	0	$\left(a_{1}^{\left(8\right)},b_{1}^{\left(1\right)}\right)$	0.65	$\left(a_{1}^{\left(8\right)},g_{1}^{\left(1\right)}\right)$	0
$\left(a_{1}^{\left(3\right)},b_{1}^{\left(2\right)}\right)$	0.35	$\left(a_{1}^{\left(3\right)},g_{1}^{\left(2\right)}\right)$	0	$\left(a_{1}^{\left(8\right)},b_{1}^{\left(2\right)}\right)$	0.55	$\left(a_{1}^{\left(8\right)},g_{1}^{\left(2\right)}\right)$	0.2
$\left(a_{1}^{\left(3\right)},b_{1}^{\left(3\right)}\right)$	0.65	$\left(a_{1}^{\left(3\right)},g_{1}^{\left(3\right)}\right)$	0	$\left(a_{1}^{\left(8\right)},b_{1}^{\left(3\right)}\right)$	0.65	$\left(a_{1}^{\left(8\right)},g_{1}^{\left(3\right)}\right)$	0
$\left(a_{1}^{\left(4\right)},b_{1}^{\left(1\right)}\right)$	0.45	$\left(a_{1}^{\left(4\right)},g_{1}^{\left(1\right)}\right)$	0	$\left(a_{1}^{\left(9\right)},b_{1}^{\left(1\right)}\right)$	0.65	$\left(a_{1}^{\left(9\right)},g_{1}^{\left(1\right)}\right)$	0
$\left(a_{1}^{\left(4\right)},b_{1}^{\left(2\right)}\right)$	0.35	$\left(a_{1}^{\left(4\right)},g_{1}^{\left(2\right)}\right)$	0	$\left(a_{1}^{\left(9\right)},b_{1}^{\left(2\right)}\right)$	0.55	$\left(a_{1}^{\left(9\right)},g_{1}^{\left(2\right)}\right)$	0.2
$\left(a_{1}^{\left(4\right)},b_{1}^{\left(3\right)}\right)$	0.65	$\left(a_{1}^{\left(4\right)},g_{1}^{\left(3\right)}\right)$	0	$\left(a_{1}^{\left(9\right)},b_{1}^{\left(3\right)}\right)$	0.65	$\left(a_{1}^{\left(9\right)},g_{1}^{\left(3\right)}\right)$	0
$\left(a_{1}^{\left(5\right)},b_{1}^{\left(1\right)}\right)$	0.65	$\left(a_{1}^{\left(5\right)},g_{1}^{\left(1\right)}\right)$	0	$\left(a_{1}^{\left(10\right)},b_{1}^{\left(1\right)}\right)$	0.65	$\left(a_{1}^{\left(10\right)},g_{1}^{\left(1\right)}\right)$	0.2
$\left(a_{1}^{\left(5\right)},b_{1}^{\left(2\right)}\right)$	0.35	$\left(a_{1}^{\left(5\right)},g_{1}^{\left(2\right)}\right)$	0.2	$\left(a_{1}^{\left(10\right)},b_{1}^{\left(2\right)}\right)$	0.55	$\left(a_{1}^{\left(10\right)},g_{1}^{\left(2\right)}\right)$	0.2
$\left(a_{1}^{\left(5\right)},b_{1}^{\left(3\right)}\right)$	0.65	$\left(a_{1}^{\left(5\right)},g_{1}^{\left(3\right)}\right)$	0	$\left(a_{1}^{\left(10\right)},b_{1}^{\left(3\right)}\right)$	0.65	$\left(a_{1}^{\left(10\right)},g_{1}^{\left(3\right)}\right)$	0
**Stage 2**							
$\left(a_{2},b_{2}\right)$	$\Psi_{2}\left(\cdot \right)$	$\left(a_{2},g_{2}\right)$	$\Psi_{2}\left(\cdot \right)$	$\left(a_{2},b_{2}\right)$	$\Psi_{2}\left(\cdot \right)$	$\left(a_{2},g_{2}\right)$	$\Psi_{2}\left(\cdot \right)$
$\left(a_{2}^{\left(1\right)},b_{2}^{\left(1\right)}\right)$	0.65	$\left(a_{2}^{\left(1\right)},g_{2}^{\left(1\right)}\right)$	0.2	$\left(a_{2}^{\left(7\right)},b_{2}^{\left(1\right)}\right)$	0.45	$\left(a_{2}^{\left(7\right)},g_{2}^{\left(1\right)}\right)$	0
$\left(a_{2}^{\left(1\right)},b_{2}^{\left(2\right)}\right)$	0.2	$\left(a_{2}^{\left(1\right)},g_{2}^{\left(2\right)}\right)$	0.2	$\left(a_{2}^{\left(7\right)},b_{2}^{\left(2\right)}\right)$	0.45	$\left(a_{2}^{\left(7\right)},g_{2}^{\left(2\right)}\right)$	0.45
$\left(a_{2}^{\left(1\right)},b_{2}^{\left(3\right)}\right)$	0.2	$\left(a_{2}^{\left(1\right)},g_{2}^{\left(3\right)}\right)$	0	$\left(a_{2}^{\left(7\right)},b_{2}^{\left(3\right)}\right)$	0.65	$\left(a_{2}^{\left(7\right)},g_{2}^{\left(3\right)}\right)$	0
$\left(a_{2}^{\left(2\right)},b_{2}^{\left(1\right)}\right)$	0.45	$\left(a_{2}^{\left(2\right)},g_{2}^{\left(1\right)}\right)$	0	$\left(a_{2}^{\left(8\right)},b_{2}^{\left(1\right)}\right)$	0.45	$\left(a_{2}^{\left(8\right)},g_{2}^{\left(1\right)}\right)$	0
$\left(a_{2}^{\left(2\right)},b_{2}^{\left(2\right)}\right)$	0.45	$\left(a_{2}^{\left(2\right)},g_{2}^{\left(2\right)}\right)$	0.45	$\left(a_{2}^{\left(8\right)},b_{2}^{\left(2\right)}\right)$	0	$\left(a_{2}^{\left(8\right)},g_{2}^{\left(2\right)}\right)$	0
$\left(a_{2}^{\left(2\right)},b_{2}^{\left(3\right)}\right)$	0.65	$\left(a_{2}^{\left(2\right)},g_{2}^{\left(3\right)}\right)$	0	$\left(a_{2}^{\left(8\right)},b_{2}^{\left(3\right)}\right)$	0.2	$\left(a_{2}^{\left(8\right)},g_{2}^{\left(3\right)}\right)$	0
$\left(a_{2}^{\left(3\right)},b_{2}^{\left(1\right)}\right)$	0.45	$\left(a_{2}^{\left(3\right)},g_{2}^{\left(1\right)}\right)$	0	$\left(a_{2}^{\left(9\right)},b_{2}^{\left(1\right)}\right)$	0.45	$\left(a_{2}^{\left(9\right)},g_{2}^{\left(1\right)}\right)$	0
$\left(a_{2}^{\left(3\right)},b_{2}^{\left(2\right)}\right)$	0.45	$\left(a_{2}^{\left(3\right)},g_{2}^{\left(2\right)}\right)$	0.45	$\left(a_{2}^{\left(9\right)},b_{2}^{\left(2\right)}\right)$	0	$\left(a_{2}^{\left(9\right)},g_{2}^{\left(2\right)}\right)$	0
$\left(a_{2}^{\left(3\right)},b_{2}^{\left(3\right)}\right)$	0.65	$\left(a_{2}^{\left(3\right)},g_{2}^{\left(3\right)}\right)$	0	$\left(a_{2}^{\left(9\right)},b_{2}^{\left(3\right)}\right)$	0.2	$\left(a_{2}^{\left(9\right)},g_{2}^{\left(3\right)}\right)$	0
$\left(a_{2}^{\left(4\right)},b_{2}^{\left(1\right)}\right)$	0.65	$\left(a_{2}^{\left(4\right)},g_{2}^{\left(1\right)}\right)$	0.2	$\left(a_{2}^{\left(10\right)},b_{2}^{\left(1\right)}\right)$	0.45	$\left(a_{2}^{\left(10\right)},g_{2}^{\left(1\right)}\right)$	0
$\left(a_{2}^{\left(4\right)},b_{2}^{\left(2\right)}\right)$	0.2	$\left(a_{2}^{\left(4\right)},g_{2}^{\left(2\right)}\right)$	0.2	$\left(a_{2}^{\left(10\right)},b_{2}^{\left(2\right)}\right)$	0.45	$\left(a_{2}^{\left(10\right)},g_{2}^{\left(2\right)}\right)$	0.45
$\left(a_{2}^{\left(4\right)},b_{2}^{\left(3\right)}\right)$	0.2	$\left(a_{2}^{\left(4\right)},g_{2}^{\left(3\right)}\right)$	0	$\left(a_{2}^{\left(10\right)},b_{2}^{\left(3\right)}\right)$	0.65	$\left(a_{2}^{\left(10\right)},g_{2}^{\left(3\right)}\right)$	0
$\left(a_{2}^{\left(5\right)},b_{2}^{\left(1\right)}\right)$	0.45	$\left(a_{2}^{\left(5\right)},g_{2}^{\left(1\right)}\right)$	0	$\left(a_{2}^{\left(11\right)},b_{2}^{\left(1\right)}\right)$	0.45	$\left(a_{2}^{\left(11\right)},g_{2}^{\left(1\right)}\right)$	0
$\left(a_{2}^{\left(5\right)},b_{2}^{\left(2\right)}\right)$	0	$\left(a_{2}^{\left(5\right)},g_{2}^{\left(2\right)}\right)$	0	$\left(a_{2}^{\left(11\right)},b_{2}^{\left(2\right)}\right)$	0	$\left(a_{2}^{\left(11\right)},g_{2}^{\left(2\right)}\right)$	0
$\left(a_{2}^{\left(5\right)},b_{2}^{\left(3\right)}\right)$	0.2	$\left(a_{2}^{\left(5\right)},g_{2}^{\left(3\right)}\right)$	0	$\left(a_{2}^{\left(11\right)},b_{2}^{\left(3\right)}\right)$	0.2	$\left(a_{2}^{\left(11\right)},g_{2}^{\left(3\right)}\right)$	0.35
$\left(a_{2}^{\left(6\right)},b_{2}^{\left(1\right)}\right)$	0.45	$\left(a_{2}^{\left(6\right)},g_{2}^{\left(1\right)}\right)$	0	$\left(a_{2}^{\left(12\right)},b_{2}^{\left(1\right)}\right)$	0.45	$\left(a_{2}^{\left(12\right)},g_{2}^{\left(1\right)}\right)$	0
$\left(a_{2}^{\left(6\right)},b_{2}^{\left(2\right)}\right)$	0.45	$\left(a_{2}^{\left(6\right)},g_{2}^{\left(2\right)}\right)$	0.45	$\left(a_{2}^{\left(12\right)},b_{2}^{\left(2\right)}\right)$	0	$\left(a_{2}^{\left(12\right)},g_{2}^{\left(2\right)}\right)$	0
$\left(a_{2}^{\left(6\right)},b_{2}^{\left(3\right)}\right)$	0.65	$\left(a_{2}^{\left(6\right)},g_{2}^{\left(3\right)}\right)$	0	$\left(a_{2}^{\left(12\right)},b_{2}^{\left(3\right)}\right)$	0.2	$\left(a_{2}^{\left(12\right)},g_{2}^{\left(3\right)}\right)$	0.35
**Stage 3**							
$\left(a_{3},b_{3}\right)$	$\Psi_{3}\left(\cdot \right)$	$\left(a_{3},g_{3}\right)$	$\Psi_{3}\left(\cdot \right)$	$\left(a_{3},b_{3}\right)$	$\Psi_{3}\left(\cdot \right)$	$\left(a_{3},g_{3}\right)$	$\Psi_{3}\left(\cdot \right)$
$\left(a_{3}^{\left(1\right)},b_{3}^{\left(1\right)}\right)$	0.35	$\left(a_{3}^{\left(1\right)},g_{3}^{\left(1\right)}\right)$	0.2	$\left(a_{3}^{\left(7\right)},b_{3}^{\left(1\right)}\right)$	1	$\left(a_{3}^{\left(7\right)},g_{3}^{\left(1\right)}\right)$	0
$\left(a_{3}^{\left(1\right)},b_{3}^{\left(2\right)}\right)$	0.55	$\left(a_{3}^{\left(1\right)},g_{3}^{\left(2\right)}\right)$	0.2	$\left(a_{3}^{\left(7\right)},b_{3}^{\left(2\right)}\right)$	0.35	$\left(a_{3}^{\left(7\right)},g_{3}^{\left(2\right)}\right)$	0.2
$\left(a_{3}^{\left(1\right)},b_{3}^{\left(3\right)}\right)$	0.55	$\left(a_{3}^{\left(1\right)},g_{3}^{\left(3\right)}\right)$	0.35	$\left(a_{3}^{\left(7\right)},b_{3}^{\left(3\right)}\right)$	0.35	$\left(a_{3}^{\left(7\right)},g_{3}^{\left(3\right)}\right)$	0.35
$\left(a_{3}^{\left(2\right)},b_{3}^{\left(1\right)}\right)$	0.35	$\left(a_{3}^{\left(2\right)},g_{3}^{\left(1\right)}\right)$	0.2	$\left(a_{3}^{\left(8\right)},b_{3}^{\left(1\right)}\right)$	0.2	$\left(a_{3}^{\left(8\right)},g_{3}^{\left(1\right)}\right)$	0.2
$\left(a_{3}^{\left(2\right)},b_{3}^{\left(2\right)}\right)$	0.55	$\left(a_{3}^{\left(2\right)},g_{3}^{\left(2\right)}\right)$	0.2	$\left(a_{3}^{\left(8\right)},b_{3}^{\left(2\right)}\right)$	0.35	$\left(a_{3}^{\left(8\right)},g_{3}^{\left(2\right)}\right)$	0.2
$\left(a_{3}^{\left(2\right)},b_{3}^{\left(3\right)}\right)$	0.2	$\left(a_{3}^{\left(2\right)},g_{3}^{\left(3\right)}\right)$	0.35	$\left(a_{3}^{\left(8\right)},b_{3}^{\left(3\right)}\right)$	0.55	$\left(a_{3}^{\left(8\right)},g_{3}^{\left(3\right)}\right)$	0
$\left(a_{3}^{\left(3\right)},b_{3}^{\left(1\right)}\right)$	0.35	$\left(a_{3}^{\left(3\right)},g_{3}^{\left(1\right)}\right)$	0	$\left(a_{3}^{\left(9\right)},b_{3}^{\left(1\right)}\right)$	0.2	$\left(a_{3}^{\left(9\right)},g_{3}^{\left(1\right)}\right)$	0.2
$\left(a_{3}^{\left(3\right)},b_{3}^{\left(2\right)}\right)$	0.35	$\left(a_{3}^{\left(3\right)},g_{3}^{\left(2\right)}\right)$	0.2	$\left(a_{3}^{\left(9\right)},b_{3}^{\left(2\right)}\right)$	0	$\left(a_{3}^{\left(9\right)},g_{3}^{\left(2\right)}\right)$	0.2
$\left(a_{3}^{\left(3\right)},b_{3}^{\left(3\right)}\right)$	0.55	$\left(a_{3}^{\left(3\right)},g_{3}^{\left(3\right)}\right)$	0.35	$\left(a_{3}^{\left(9\right)},b_{3}^{\left(3\right)}\right)$	0.55	$\left(a_{3}^{\left(9\right)},g_{3}^{\left(3\right)}\right)$	0
$\left(a_{3}^{\left(4\right)},b_{3}^{\left(1\right)}\right)$	0.35	$\left(a_{3}^{\left(4\right)},g_{3}^{\left(1\right)}\right)$	0.2	$\left(a_{3}^{\left(10\right)},b_{3}^{\left(1\right)}\right)$	0.2	$\left(a_{3}^{\left(10\right)},g_{3}^{\left(1\right)}\right)$	0
$\left(a_{3}^{\left(4\right)},b_{3}^{\left(2\right)}\right)$	0.55	$\left(a_{3}^{\left(4\right)},g_{3}^{\left(2\right)}\right)$	0.2	$\left(a_{3}^{\left(10\right)},b_{3}^{\left(2\right)}\right)$	0.35	$\left(a_{3}^{\left(10\right)},g_{3}^{\left(2\right)}\right)$	0
$\left(a_{3}^{\left(4\right)},b_{3}^{\left(3\right)}\right)$	0.2	$\left(a_{3}^{\left(4\right)},g_{3}^{\left(3\right)}\right)$	0.35	$\left(a_{3}^{\left(10\right)},b_{3}^{\left(3\right)}\right)$	0.35	$\left(a_{3}^{\left(10\right)},g_{3}^{\left(3\right)}\right)$	0.8
$\left(a_{3}^{\left(5\right)},b_{3}^{\left(1\right)}\right)$	0.35	$\left(a_{3}^{\left(5\right)},g_{3}^{\left(1\right)}\right)$	0.2	$\left(a_{3}^{\left(11\right)},b_{3}^{\left(1\right)}\right)$	0.2	$\left(a_{3}^{\left(11\right)},g_{3}^{\left(1\right)}\right)$	0
$\left(a_{3}^{\left(5\right)},b_{3}^{\left(2\right)}\right)$	0.55	$\left(a_{3}^{\left(5\right)},g_{3}^{\left(2\right)}\right)$	0.2	$\left(a_{3}^{\left(11\right)},b_{3}^{\left(2\right)}\right)$	0.35	$\left(a_{3}^{\left(11\right)},g_{3}^{\left(2\right)}\right)$	0.2
$\left(a_{3}^{\left(5\right)},b_{3}^{\left(3\right)}\right)$	0.2	$\left(a_{3}^{\left(5\right)},g_{3}^{\left(3\right)}\right)$	0	$\left(a_{3}^{\left(11\right)},b_{3}^{\left(3\right)}\right)$	0.35	$\left(a_{3}^{\left(11\right)},g_{3}^{\left(3\right)}\right)$	0.45
$\left(a_{3}^{\left(6\right)},b_{3}^{\left(1\right)}\right)$	0.35	$\left(a_{3}^{\left(6\right)},g_{3}^{\left(1\right)}\right)$	0	$\left(a_{3}^{\left(12\right)},b_{3}^{\left(1\right)}\right)$	0.2	$\left(a_{3}^{\left(12\right)},g_{3}^{\left(1\right)}\right)$	0.2
$\left(a_{3}^{\left(6\right)},b_{3}^{\left(2\right)}\right)$	0.35	$\left(a_{3}^{\left(6\right)},g_{3}^{\left(2\right)}\right)$	0	$\left(a_{3}^{\left(12\right)},b_{3}^{\left(2\right)}\right)$	0	$\left(a_{3}^{\left(12\right)},g_{3}^{\left(2\right)}\right)$	0.2
$\left(a_{3}^{\left(6\right)},b_{3}^{\left(3\right)}\right)$	0.55	$\left(a_{3}^{\left(6\right)},g_{3}^{\left(3\right)}\right)$	0.35	$\left(a_{3}^{\left(12\right)},b_{3}^{\left(3\right)}\right)$	0.55	$\left(a_{3}^{\left(12\right)},g_{3}^{\left(3\right)}\right)$	0.45

**Table A9:** Values of $\Phi_{B_{t}}\left(a_{t}^{\left(i\right)}\right)$ and $\Phi_{G_{t}}\left(a_{t}^{\left(i\right)}\right)$.

Stage 1			Stage 2			Stage 3		
$a_{1}$	$\Phi_{B_{1}}\left(a_{1}^{\left(i\right)}\right)$	$\Phi_{G_{1}}\left(a_{1}^{\left(i\right)}\right)$	$a_{2}$	$\Phi_{B_{2}}\left(a_{1}^{\left(i\right)}\right)$	$\Phi_{G_{2}}\left(a_{2}^{\left(i\right)}\right)$	$a_{3}$	$\Phi_{B_{3}}\left(a_{3}^{\left(i\right)}\right)$	$\Phi_{G_{3}}\left(a_{3}^{\left(i\right)}\right)$
$a_{1}^{\left(1\right)}$	0.27	−0.7	$a_{2}^{\left(1\right)}$	−0.3	−0.7	$a_{3}^{\left(1\right)}$	−0.0333	−0.5
$a_{1}^{\left(2\right)}$	−0	−1	$a_{2}^{\left(2\right)}$	0.03	−0.7	$a_{3}^{\left(2\right)}$	−0.2667	−0.5
$a_{1}^{\left(3\right)}$	−0	−1	$a_{2}^{\left(3\right)}$	0.03	−0.7	$a_{3}^{\left(3\right)}$	−0.1667	−0.6333
$a_{1}^{\left(4\right)}$	−0	−1	$a_{2}^{\left(4\right)}$	−0.3	−0.7	$a_{3}^{\left(4\right)}$	−0.2667	−0.5
$a_{1}^{\left(5\right)}$	0.1	−0.87	$a_{2}^{\left(5\right)}$	−0.6	−1	$a_{3}^{\left(5\right)}$	−0.2667	−0.7333
$a_{1}^{\left(6\right)}$	0.1	−0.87	$a_{2}^{\left(6\right)}$	0.03	−0.7	$a_{3}^{\left(6\right)}$	−0.1667	−0.7667
$a_{1}^{\left(7\right)}$	0.1	−0.87	$a_{2}^{\left(7\right)}$	0.03	−0.7	$a_{3}^{\left(7\right)}$	−0.4	−0.6333
$a_{1}^{\left(8\right)}$	0.23	−0.87	$a_{2}^{\left(8\right)}$	−0.6	−1	$a_{3}^{\left(8\right)}$	−0.2667	−0.7333
$a_{1}^{\left(9\right)}$	0.23	−0.87	$a_{2}^{\left(9\right)}$	−0.6	−1	$a_{3}^{\left(9\right)}$	−0.5	−0.7333
$a_{1}^{\left(10\right)}$	0.23	−0.73	$a_{2}^{\left(10\right)}$	0.03	−0.7	$a_{3}^{\left(10\right)}$	−0.4	−0.4667
			$a_{2}^{\left(11\right)}$	−0.6	−0.8	$a_{3}^{\left(11\right)}$	−0.4	−0.5667
			$a_{2}^{\left(12\right)}$	−0.6	−0.8	$a_{3}^{\left(12\right)}$	−0.5	−0.4333
